# Supplementary material for: Prediction of type 2 diabetes risk in people with non-diabetic hyperglycaemia: model derivation and validation using UK primary care data
Source: BMJ Open. 2020 Oct 23;10(10):e037937. doi: 10.1136/bmjopen-2020-037937 (PMC7590356; doi:10.1136/bmjopen-2020-037937)
Supplement: Supplementary data [file bmjopen-2020-037937supp005.pdf]

**Supplementary Table S1.** Number of practices by region in total and included in the development and validation datasets.

| Practice region | Total | Dataset | |
|---|---|---|---|
| | | Development | Validation |
| North East | 11 | 8 | 3 |
| North West | 85 | 60 | 26 |
| Yorkshire & The Humber | 28 | 20 | 8 |
| East Midlands | 25 | 18 | 8 |
| West Midlands | 61 | 43 | 18 |
| East of England | 54 | 38 | 16 |
| South West | 61 | 43 | 18 |
| South Central | 56 | 39 | 17 |
| London | 95 | 67 | 29 |
| South East Coast | 68 | 48 | 20 |
| Northern Ireland | 25 | 18 | 8 |
| Scotland | 94 | 66 | 28 |
| Wales | 77 | 54 | 23 |
| **Total** | **740** | **518** | **222** |
