# Supplementary material for: Prediction of type 2 diabetes risk in people with non-diabetic hyperglycaemia: model derivation and validation using UK primary care data
Source: BMJ Open. 2020 Oct 23;10(10):e037937. doi: 10.1136/bmjopen-2020-037937 (PMC7590356; doi:10.1136/bmjopen-2020-037937)
Supplement: Supplementary data [file bmjopen-2020-037937supp006.pdf]

Supplementary Table S2. Percent of patients missing potential predictor variables.

Predictor variable	Missing n	Missing %
Waist circumference	153,592	99.3
Liver function test	153,493	99.2
Pulse rate	133,890	86.5
BMI	113,840	73.6
Index of Multiple Deprivation	63,524	41.1
Systolic blood pressure	48,390	31.3
Diastolic blood pressure	48,390	31.3
Ethnicity	41,121	26.6
Serum cholesterol	38,910	25.2
HbA1c	0	0
Age	0	0
Sex	0	0
Current alcohol use	0	0
Current smoker	0	0
Antihypertensives	0	0
Atypical antipsychotics	0	0
Aspirin	0	0
Corticosteroids	0	0
Statins	0	0
Bipolar disease or schizophrenia	0	0
Cardiovascular disease	0	0
Depression	0	0
Learning disability	0	0
Diabetes in family	0	0
Polycystic ovarian syndrome	0	0
Gestational diabetes	0	0
Renal/kidney disease	0	0
Sleep apnoea	0	0
