# Supplementary material for: Prediction of type 2 diabetes risk in people with non-diabetic hyperglycaemia: model derivation and validation using UK primary care data
Source: BMJ Open. 2020 Oct 23;10(10):e037937. doi: 10.1136/bmjopen-2020-037937 (PMC7590356; doi:10.1136/bmjopen-2020-037937)
Supplement: Supplementary data [file bmjopen-2020-037937supp007.pdf]

Supplementary Table S3. Incidence of Type 2 diabetes per 1,000 person years with 95% confidence intervals.

		Total			Dataset: Development			Dataset: Validation		
		Py	n	Rate (95% CI)	Py	n	Rate (95% CI)	Py	n	Rate (95% CI)
Total		408,350.5	13,115	32.1 (31.6-32.7)	293,237.8	9,332	31.8 (31.2-32.5)	115,112.6	3,783	32.9 (31.8-33.9)
Age group	<30	4,285.1	79	18.4 (14.8-23.0)	3,017.0	56	18.6 (14.3-24.1)	1,268.1	23	18.1 (12.1-27.3)
	30-39	15,214.7	307	20.2 (18.0-22.6)	11,050.8	231	20.9 (18.4-23.8)	4,164.0	76	18.3 (14.6-22.9)
	40-49	43,354.3	1,157	26.7 (25.2-28.3)	31,539.3	836	26.5 (24.8-28.4)	11,815.0	321	27.2 (24.4-30.3)
	50-59	81,437.4	2,399	29.5 (28.3-30.7)	58,691.3	1,730	29.5 (28.1-30.9)	22,746.1	669	29.4 (27.3-31.7)
	60-69	109,599.6	3,808	34.7 (33.7-35.9)	79,177.3	2,709	34.2 (32.9-35.5)	30,422.4	1,099	36.1 (34.1-38.3)
	70-79	96,100.4	3,553	37.0 (35.8-38.2)	68,493.3	2,527	36.9 (35.5-38.4)	27,607.1	1,026	37.2 (35.0-39.5)
	80-89	50,818.9	1,629	32.1 (30.5-33.7)	36,072.2	1,114	30.9 (29.1-32.8)	14,746.7	515	34.9 (32.0-38.1)
	90+	7,540.0	183	24.3 (21.0-28.1)	5,196.7	129	24.8 (20.9-29.5)	2,343.2	54	23.0 (17.6-30.1)
Sex	Male	186,953.5	6,612	35.4 (34.5-36.2)	134,390.2	4,719	35.1 (34.1-36.1)	52,563.3	1,893	36.0 (34.4-37.7)
	Female	221,397.0	6,503	29.4 (28.7-30.1)	158,847.6	4,613	29.0 (28.2-29.9)	62,549.3	1,890	30.2 (28.9-31.6)
Ethnicity	Non-white	38,606.0	1,154	29.9 (28.2-31.7)	29,281.3	863	29.5 (27.6-31.5)	9,324.7	291	31.2 (27.8-35.0)
	White	257,231.3	8,446	32.8 (32.1-33.5)	181,622.3	5,878	32.4 (31.5-33.2)	75,609.0	2,568	34.0 (32.7-35.3)
Current alcohol user	No	321,672.8	10,049	31.2 (30.6-31.9)	231,489.6	7,223	31.2 (30.5-31.9)	90,183.2	2,826	31.3 (30.2-32.5)
	Yes	86,677.6	3,066	35.4 (34.1-36.6)	61,748.2	2,109	34.2 (32.7-35.6)	24,929.4	957	38.4 (36.0-40.9)
Current smoker	No	351,866.5	11,355	32.3 (31.7-32.9)	252,907.8	8,103	32.0 (31.3-32.7)	98,958.7	3,252	32.9 (31.8-34.0)
	Yes	56,483.9	1,760	31.2 (29.7-32.6)	40,330.0	1,229	30.5 (28.8-32.2)	16,154.0	531	32.9 (30.2-35.8)
Antihypertensives	No	402,244.5	12,840	31.9 (31.4-32.5)	288,800.1	9,137	31.6 (31.0-32.3)	113,444.4	3,703	32.6 (31.6-33.7)
	Yes	6,105.9	275	45.0 (40.0-50.7)	4,437.7	195	43.9 (38.2-50.6)	1,668.3	80	48.0 (38.5-59.7)
Atypical antipsychotics	No	397,003.1	12,760	32.1 (31.6-32.7)	284,987.3	9,084	31.9 (31.2-32.5)	112,015.8	3,676	32.8 (31.8-33.9)
	Yes	11,347.4	355	31.3 (28.2-34.7)	8,250.5	248	30.1 (26.5-34.0)	3,096.9	107	34.6 (28.6-41.8)
Aspirin	No	282,265.5	7,971	28.2 (27.6-28.9)	202,397.8	5,686	28.1 (27.4-28.8)	79,867.7	2,285	28.6 (27.5-29.8)
	Yes	126,085.0	5,144	40.8 (39.7-41.9)	90,840.0	3,646	40.1 (38.9-41.5)	35,245.0	1,498	42.5 (40.4-44.7)
Corticosteroids	No	132,237.8	3,781	28.6 (27.7-29.5)	94,557.1	2,721	28.8 (27.7-29.9)	37,680.7	1,060	28.1 (26.5-29.9)
	Yes	276,112.7	9,334	33.8 (33.1-34.5)	198,680.8	6,611	33.3 (32.5-34.1)	77,431.9	2,723	35.2 (33.9-36.5)
Statins	No	197,618.7	4,184	21.2 (20.5-21.8)	141,932.3	2,977	21.0 (20.2-21.7)	55,686.3	1,207	21.7 (20.5-22.9)
	Yes	210,731.8	8,931	42.4 (41.5-43.3)	151,305.5	6,355	42.0 (41.0-43.0)	59,426.3	2,576	43.3 (41.7-45.1)
Schizophrenia/bipolar	No	402,889.4	12,937	32.1 (31.6-32.7)	289,246.4	9,212	31.8 (31.2-32.5)	113,642.9	3,725	32.8 (31.7-33.8)
	Yes	5,461.1	178	32.6 (28.1-37.8)	3,991.4	120	30.1 (25.1-36.0)	1,469.7	58	39.5 (30.5-51.0)
Cardiovascular disease	No	361,574.5	11,297	31.2 (30.7-31.8)	260,237.0	8,074	31.0 (30.4-31.7)	101,337.5	3,223	31.8 (30.7-32.9)
	Yes	46,776.0	1,818	38.9 (37.1-40.7)	33,000.8	1,258	38.1 (36.1-40.3)	13,775.1	560	40.7 (37.4-44.2)
Depression	No	303,786.2	9,875	32.5 (31.9-33.2)	219,040.2	7,043	32.2 (31.4-32.9)	84,746.0	2,832	33.4 (32.2-34.7)

	Yes	104,564.3	3,240	31.0 (29.9-32.1)	74,197.7	2,289	30.9 (29.6-32.1)	30,366.6	951	31.3 (29.4-33.4)
Learning disability	No	406,734.9	13,060	32.1 (31.6-32.7)	292,204.8	9,301	31.8 (31.2-32.5)	114,530.1	3,759	32.8 (31.8-33.9)
	Yes	1,615.6	55	34.0 (26.1-44.3)	1,033.0	31	30.0 (21.1-42.7)	582.5	24	41.2 (27.6-61.5)
Diabetes in family	No	407,867.0	13,091	32.1 (31.6-32.7)	292,821.8	9,311	31.8 (31.2-32.4)	115,045.1	3,780	32.9 (31.8-33.9)
	Yes	483.5	24	49.6 (33.3-74.1)	416.0	21	50.5 (32.9-77.4)	67.5	<5	44.4 (14.3-137.8)
Renal/kidney disease	No	368,309.2	11,766	31.9 (31.4-32.5)	265,292.2	8,403	31.7 (31.0-32.4)	103,016.9	3,363	32.6 (31.6-33.8)
	Yes	40,041.3	1,349	33.7 (31.9-35.5)	27,945.6	929	33.2 (31.2-35.5)	12,095.7	420	34.7 (31.6-38.2)
Sleep apnoea	No	403,300.5	12,896	32.0 (31.4-32.5)	289,628.0	9,178	31.7 (31.0-32.3)	113,672.4	3,718	32.7 (31.7-33.8)
	Yes	5,050.0	219	43.4 (38.0-49.5)	3,609.8	154	42.7 (36.4-50.0)	1,440.2	65	45.1 (35.4-57.6)
PCOS*	No	219,461.5	6,441	29.3 (28.6-30.1)	157,488.6	4,571	29.0 (28.2-29.9)	61,972.9	1,870	30.2 (28.8-31.6)
	Yes	1,935.5	62	32.0 (25.0-41.1)	1,359.0	42	30.9 (22.8-41.8)	576.5	20	34.7 (22.4-53.8)
Gestational diabetes*	No	219,205.1	6,423	29.3 (28.6-30.0)	157,163.0	4,550	29.0 (28.1-29.8)	62,042.1	1,873	30.2 (28.9-31.6)
	Yes	2,191.9	80	36.5 (29.3-45.4)	1,684.7	63	37.4 (29.2-47.9)	507.2	17	33.5 (20.8-53.9)
HbA1c (mmol/mol)	42	143,564.4	2,341	16.3 (15.7-17.0)	102,303.4	1,650	16.1 (15.4-16.9)	41,261.0	691	16.7 (15.5-18.0)
	43	103,706.7	2,496	24.1 (23.1-25.0)	74,289.4	1,762	23.7 (22.6-24.9)	29,417.3	734	25.0 (23.2-26.8)
	44	72,839.5	2,563	35.2 (33.9-36.6)	52,495.5	1,801	34.3 (32.8-35.9)	20,344.0	762	37.5 (34.9-40.2)
	45	48,523.8	2,407	49.6 (47.7-51.6)	35,497.9	1,724	48.6 (46.3-50.9)	13,025.9	683	52.4 (48.6-56.5)
	46	31,687.9	2,473	78.0 (75.0-81.2)	22,985.0	1,794	78.1 (74.5-81.7)	8,702.9	679	78.0 (72.4-84.1)
	47	8,028.2	835	104.0 (97.2-111.3)	5,666.6	601	106.1 (97.9-114.9)	2,361.6	234	99.1 (87.2-112.6)
Cholesterol (mmol/L)	<5.0	130,946.3	4,568	34.9 (33.9-35.9)	93,611.8	3,273	35.0 (33.8-36.2)	37,334.5	1,295	34.7 (32.8-36.6)
	5.0-6.9	152,342.9	4,978	32.7 (31.8-33.6)	109,313.4	3,548	32.5 (31.4-33.5)	43,029.5	1,430	33.2 (31.6-35.0)
	≥7.0	24,848.2	817	32.9 (30.7-35.2)	17,982.7	573	31.9 (29.4-34.6)	6,865.5	244	35.5 (31.3-40.3)
Systolic BP (mmHg)	<140	147,766.9	4,476	30.3 (29.4-31.2)	105,813.8	3,195	30.2 (29.2-31.3)	41,953.1	1,281	30.5 (28.9-32.3)
	≥140	135,710.1	5,206	38.4 (37.3-39.4)	97,560.5	3,685	37.8 (36.6-39.0)	38,149.6	1,521	39.9 (37.9-41.9)
Diastolic BP (mmHg)	<90	228,884.2	7,540	32.9 (32.2-33.7)	164,236.7	5,367	32.7 (31.8-33.6)	64,647.6	2,173	33.6 (32.2-35.1)
	≥90	54,592.8	2,142	39.2 (37.6-40.9)	39,137.6	1,513	38.7 (36.8-40.7)	15,455.2	629	40.7 (37.6-44.0)

BP=blood pressure. PCOS=Polycystic ovarian syndrome.
Table includes observed values only. Imputed values for ethnicity, serum cholesterol, and systolic and diastolic blood pressure are not included.
Index of multiple deprivation, BMI, pulse, liver function test, and waist circumference are not included in the table since these measures are not available for >33.3% of the cohort.
Age was collapsed into 10-year groups. HbA1c was collapsed into one mmol/mol increments. Cholesterol was collapsed into three clinically relevant groups (<5.0, 5.0-6.9, and ≥7.0mmol/L). Systolic blood pressure was collapsed into two clinically relevant groups based on NICE guidelines for hypertension (<140 and ≥140 mmHg) as was diastolic blood pressure (<90 and ≥90 mmHg) (27).
***The incidence was calculated among females only.**
+Note, n<5 cannot be published.
