# Supplementary material for: Prediction of type 2 diabetes risk in people with non-diabetic hyperglycaemia: model derivation and validation using UK primary care data
Source: BMJ Open. 2020 Oct 23;10(10):e037937. doi: 10.1136/bmjopen-2020-037937 (PMC7590356; doi:10.1136/bmjopen-2020-037937)
Supplement: Supplementary data [file bmjopen-2020-037937supp008.pdf]

Supplementary Table S4. Descriptive statistics for cholesterol, blood pressure, and ethnicity for observed (non-missing) development data and final development data (including observed and imputed).

Variable	Dataset	Mean	Std. Dev.
Cholesterol (mmol/L)	Observed	5.23	1.19
	Observed+Imputed	5.26	1.19
Systolic blood pressure (mmHg)	Observed	138.02	18.57
	Observed+Imputed	137.80	18.59
Diastolic blood pressure (mmHg)	Observed	79.92	11.01
	Observed+Imputed	80.21	11.01
White Ethnicity (proportion)	Observed	0.87	0.34
	Observed+Imputed	0.87	0.33

Observed+Imputed comprises the final data. The distribution of the observed data with the observed+imputed data overlaid was visually examined and no large differences were seen.
