# Supplementary material for: Prediction of type 2 diabetes risk in people with non-diabetic hyperglycaemia: model derivation and validation using UK primary care data
Source: BMJ Open. 2020 Oct 23;10(10):e037937. doi: 10.1136/bmjopen-2020-037937 (PMC7590356; doi:10.1136/bmjopen-2020-037937)

Supplementary Figure S3. Calibration plots by 10-year Type 2 diabetes risk deciles for the male and female models in one of the imputed validation datasets.

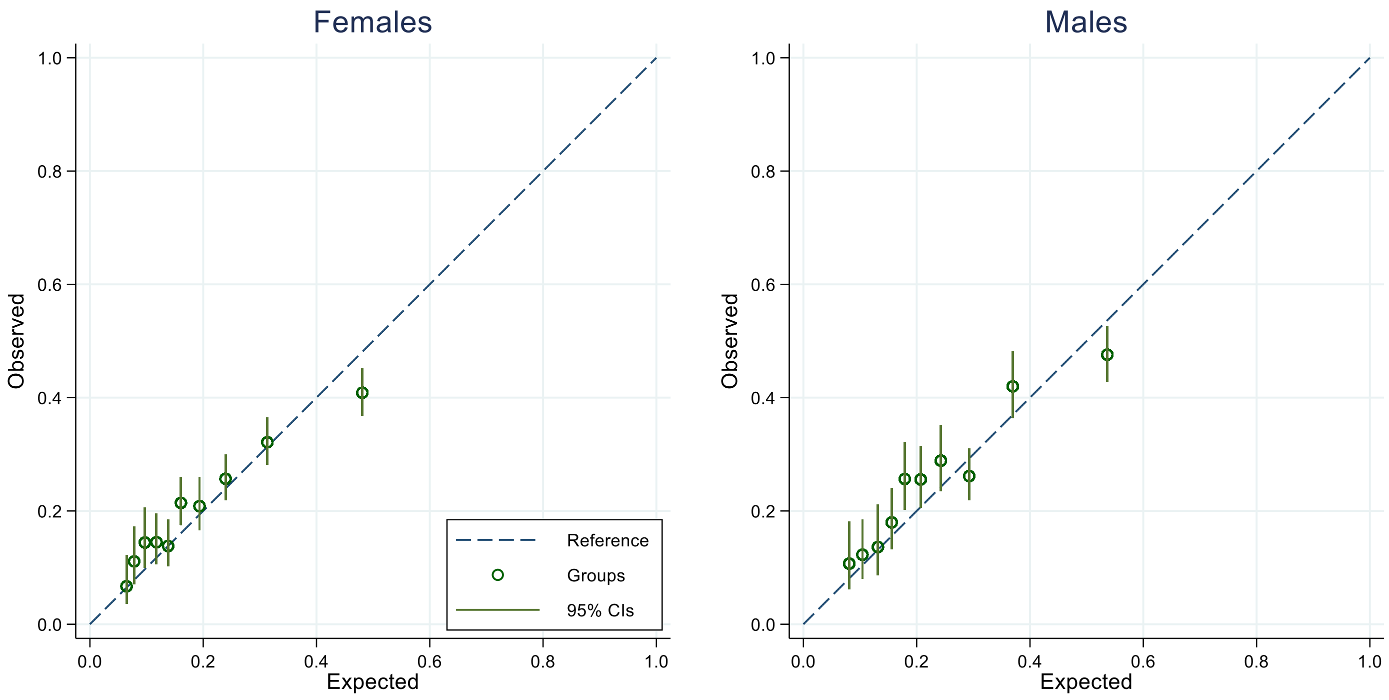

Supplement: Supplementary data [file bmjopen-2020-037937supp003.pdf]
