# Supplementary material for: Prediction of type 2 diabetes risk in people with non-diabetic hyperglycaemia: model derivation and validation using UK primary care data
Source: BMJ Open. 2020 Oct 23;10(10):e037937. doi: 10.1136/bmjopen-2020-037937 (PMC7590356; doi:10.1136/bmjopen-2020-037937)
Supplement: Author's manuscript [file bmjopen-2020-037937.draft_revisions.pdf]

BMJ Open is committed to open peer review. As part of this commitment we make the peer review history of every article we publish publicly available.

When an article is published we post the peer reviewers' comments and the authors' responses online. We also post the versions of the paper that were used during peer review. These are the versions that the peer review comments apply to.

The versions of the paper that follow are the versions that were submitted during the peer review process. They are not the versions of record or the final published versions. They should not be cited or distributed as the published version of this manuscript.

BMJ Open is an open access journal and the full, final, typeset and author-corrected version of record of the manuscript is available on our site with no access controls, subscription charges or pay-per-view fees (http://bmjopen.bmj.com).

If you have any questions on BMJ Open's open peer review process please email info.bmjopen@bmj.com

BMJ Open

Prediction of Type 2 diabetes risk in people with non-diabetic hyperglycaemia: model derivation and validation using UK primary care data

Journal:	*BMJ Open*
Manuscript ID	bmjopen-2020-037937
Article Type:	Original research
Date Submitted by the Author:	24-Feb-2020

Complete List of Authors:	Coles, Briana; University of Leicester,<br>Khunti, Kamlesh; University of Leicester, Department of Health Sciences<br>Booth, Sarah; University of Leicester, Department of Health Sciences<br>Zaccardi, Francesco; University of Leicester, Diabetes Research Centre<br>Davies, Melanie; University of Leicester, Diabetes Research Centre<br>Gray, Laura; University of Leicester, Department of Health Sciences

Keywords:	PUBLIC HEALTH, General diabetes < DIABETES & ENDOCRINOLOGY, PRIMARY CARE

SCHOLARONE™ Manuscripts

I, the Submitting Author has the right to grant and does grant on behalf of all authors of the Work (as defined in the below author licence), an exclusive licence and/or a non-exclusive licence for contributions from authors who are: i) UK Crown employees; ii) where BMJ has agreed a CC-BY licence shall apply, and/or iii) in accordance with the terms applicable for US Federal Government officers or employees acting as part of their official duties; on a worldwide, perpetual, irrevocable, royalty-free basis to BMJ Publishing Group Ltd ("BMJ") its licensees and where the relevant Journal is co-owned by BMJ to the co-owners of the Journal, to publish the Work in this journal and any other BMJ products and to exploit all rights, as set out in our licence.

The Submitting Author accepts and understands that any supply made under these terms is made by BMJ to the Submitting Author unless you are acting as an employee on behalf of your employer or a postgraduate student of an affiliated institution which is paying any applicable article publishing charge ("APC") for Open Access articles. Where the Submitting Author wishes to make the Work available on an Open Access basis (and intends to pay the relevant APC), the terms of reuse of such Open Access shall be governed by a Creative Commons licence – details of these licences and which Creative Commons licence will apply to this Work are set out in our licence referred to above.

Other than as permitted in any relevant BMJ Author's Self Archiving Policies, I confirm this Work has not been accepted for publication elsewhere, is not being considered for publication elsewhere and does not duplicate material already published. I confirm all authors consent to publication of this Work and authorise the granting of this licence.

For peer review only

Prediction of Type 2 diabetes risk in people with non-diabetic hyperglycaemia: model derivation and validation using UK primary care data

Short running title

Type 2 diabetes risk prediction in people with non-diabetic hyperglycaemia

Authors

Briana Coles[1,3], Kamlesh Khunti[1,3], Sarah Booth[2], Francesco Zaccardi [1,3], Melanie J Davies[3], Laura J Gray[2]

Affiliations

[1]Leicester Real World Evidence Unit, Diabetes Research Centre, University of Leicester, Leicester, UK.

[2]Department of Health Sciences, University of Leicester, Leicester, UK.

[3]Diabetes Research Centre, University of Leicester, Leicester General Hospital, Leicester, UK.

Corresponding author

Briana Coles, bc188@leicester.ac.uk

Gwendolen Rd, Leicester LE5 4PW, UK

Tel: +44 (0) 734 213 7177

Email: bc188@le.ac.uk

Word count

Abstract: 272

Main Text: 4,340

Tables 4; RECORD checklist; TRIPOD checklist; ISAC application; Supplementary Tables 3; Supplementary Figures 4

Keywords

Type 2 Diabetes Mellitus; Prediabetic State; Hyperglycaemia; Preventive Medicine; Preventive Health Services; Evidence-Based Practice; Risk prediction; biostatistics

For peer review only

For peer review only - http://bmjopen.bmj.com/site/about/guidelines.xhtml

ABSTRACT

Objective: Using primary care data, develop and validate sex-specific prognostic models that estimate the ten year risk of people with non-diabetic hyperglycaemia developing Type 2 diabetes.

Design: Retrospective cohort study

Setting: Primary care

Participants: 154,705 adult patients with non-diabetic hyperglycaemia

Primary outcome: Development of type 2 diabetes

Methods: This study used data routinely collected in UK primary care from general practices contributing to the Clinical Practice Research Datalink. Patients were split into development (n=109,077) and validation datasets (n=45,628). Potential predictor variables- including demographic and lifestyle factors, medical and family history, prescribed medications, and clinical measures- were included in survival models following the imputation of missing data. Measures of calibration at 10 years and discrimination were determined using the validation dataset.

Results: In the development dataset, 9,332 patients developed Type 2 diabetes during 293,238 person-years of follow-up (31.8 per 1,000 person-years). In the validation dataset, 3,783 patients developed Type 2 diabetes during 115,113 person-years of follow-up (32.9 per 1,000 person-years). The final prognostic models comprised 14 and 16 predictor variables for males and females, respectively. Both models had good calibration and high levels of discrimination. The performance statistics for the male model were: Harrell's C statistic of 0.700 in the development and 0.701 in the validation dataset, with a calibration slope of 0.974 in the validation dataset. For the female model, Harrell's C statistics were 0.720 and 0.718, respectively, while the calibration slope was 0.994 in the validation dataset.

For peer review only - http://bmjopen.bmj.com/site/about/guidelines.xhtml

Conclusion: These models could be used in primary care to identify those with non-diabetic hyperglycaemia most at risk of developing Type 2 diabetes for targeted referral to the National Health Service Diabetes Prevention Programme.

For peer review only

For peer review only - http://bmjopen.bmj.com/site/about/guidelines.xhtml

ARTICLE SUMMARY

Strengths

- A large, representative primary care database was used to develop the models using HbA1c to quantify blood glucose.
- A range of predictors were considered specifically selected due to clinical relevance to development of Type 2 diabetes.

Limitations

- The cohort was split into development and validation datasets instead of using a fully external database to validate the model, but given the size of the cohort and the large number of events, this likely had little effect on model development.
- The outcome for this study was defined using a single medcode or test result indicating Type 2 diabetes.

For peer review only

For peer review only - http://bmjopen.bmj.com/site/about/guidelines.xhtml

INTRODUCTION

People with blood glucose levels raised beyond normal but not high enough for a formal diagnosis of Type 2 diabetes (i.e. HbA1c 6.0-6.4% or 42-47 mmol/mol) are at high risk of eventually developing Type 2 diabetes. This high risk state has been termed non-diabetic hyperglycaemia (NDH) or prediabetes (1). In 2015 in England it was estimated that there were five million people aged 16 years and over with NDH, a prevalence of 11.4% (1). The prevalence was much lower in people younger than 40 years of age, with the exception of minority ethnic populations (1). Evidence from large-scale clinical trials has shown that the development of Type 2 diabetes can be delayed or even prevented if those with NDH are enrolled into a diabetes prevention programme (2, 3).

Diabetes prevention programmes encourage participants to change their behaviour with a focus on increasing physical activity, improving diet quality and reducing weight. These programmes have been developed and tested internationally (2, 4-6). Initially studies focused on very intensive programmes – for example a programme developed and tested within the US involved 16 one to one individualised sessions over six months, followed by monthly individual and group based sessions to reinforce messages (4). The trial found a 58% reduction in the risk of Type 2 diabetes in those randomised to receive the prevention programme compared to standard care. Other studies conducted in Finland and China with similar programmes found comparable results (5, 6). Such resource intensive programmes, although very effective, are not viable for delivery within an NHS setting.

Therefore, emphasis shifted to developing a more pragmatic programme that could be delivered in a group setting and requires less contact time. The National Health Service's Diabetes Prevention Programme (NHS DPP) launched in 2016 and is open to adults with NDH (7, 8). The NHS estimates that once the NHS DPP is fully rolled out in 2020, 100,000 people will access the programme each year (9). Based on this, it will take over 50 years for all those with NDH to access the programme.

For peer review only - http://bmjopen.bmj.com/site/about/guidelines.xhtml

Many prognostic and diagnostic models have been developed and validated for identifying those with undiagnosed Type 2 diabetes, NDH or those at risk of developing Type 2 diabetes (10-12). Evidence shows that the risk of developing Type 2 diabetes in those with NDH is variable. Some people with NDH will revert to normal glucose levels over time, with only a subset going on to develop Type 2 diabetes (13). In the era of big data and personalised medicine, utilising data stored in primary care to target referrals to those at highest risk may be a more efficient use of the NHS DPP than the current blanket referral approach.

To date no validated risk assessments for use in those with NDH have been developed for use in the UK. Therefore, we developed and validated sex-specific prognostic models to quantify the 10-year risk of those with NDH developing Type 2 diabetes using data routinely collected in primary care. Such models should be used to target referrals to the NHS DPP.

For peer review only

For peer review only - http://bmjopen.bmj.com/site/about/guidelines.xhtml

METHODS

Study design and data source

This observational retrospective cohort study included a sample of primary care patients from the UK who were registered with practices contributing to the Clinical Practice Research Datalink (CPRD). The CPRD includes anonymised primary care electronic health records for over 11.3 million patients from 674 UK practices dating back to 1987 (14). The CPRD includes data for approximately 6.9% of the UK population and is broadly representative of the age, sex and ethnicity of the UK general population (14). When available, patients were also linked to Office of National Statistics (ONS) to obtain the date of death and Hospital Episode Statistics (HES) to obtain ethnicity (both available for 59% of patients in the study cohort). Linked Index of Multiple Deprivation data (quintiles) were also obtained. Approval by the CPRD Independent Scientific Advisory Committee was granted for this study (approved protocol number 18_238).

This study included an open cohort of patients registered in CPRD aged 18 years or older with NDH. NDH was defined as an HbA1c measure within 42-47 mmol/mol (6.0-6.4%). For each patient, the index date was defined as the first recorded test measurement indicating NDH between January 1, 2000 and December 31, 2017. Patients with a diagnosis of Type 2 or Type 1 diabetes before the index date were excluded. Patients with an HbA1c measure greater than 47 mmol/mol (6.4%) before the index date were also excluded as these patients were assumed to be in the process of confirming a diagnosis of Type 2 diabetes. Patients prescribed metformin, the current first line therapy for Type 2 diabetes, were also excluded. Patients were followed up for a maximum of 10 years until diagnosis of Type 2 diabetes, or censoring (transferring out of practice, death, or the end of study on December 31, 2017, whichever came first).

For peer review only - http://bmjopen.bmj.com/site/about/guidelines.xhtml

The cohort was split into a development and validation dataset. To split the cohort, practices of registration were stratified by region and patients were clustered by practice (Supplementary Table S1). Approximately 33% of practices in each region were randomly assigned to the validation dataset.

Sample size

There were 71,063 males and 83,642 females meeting the inclusion criteria (Supplementary Figure S1). This resulted in 50,049 males and 59,028 females in the development dataset and 21,014 males and 24,614 females in the validation dataset. Within the development dataset, 4,719 males and 4,613 females developed Type 2 diabetes. Riley *et al.* have proposed an approach for calculating the minimum number of events per predictor parameter for a survival model based on the model's anticipated R squared, event rate, follow up time and number of predictor parameters (15). We used the R squared, event rate, and mean follow up for men and women from a similar study to estimate the required sample size.(16) For women, based on 31 predictor parameters (deprivation has five categories) considered for our study, the required minimum sample size was 3,406. For men, based on 29 predictor parameters considered for our study, the required minimum sample size was 2,585.

Outcome

The outcome was the first diagnosis of Type 2 diabetes recorded within the CPRD between January 1, 2000 and December 31, 2017. The first diagnosis of Type 2 diabetes was identified by medcode; HbA1c measure greater than 47 mmol/mol (6.4%); random blood glucose measure greater than 11.0 mmol/L (199 mg/dL); or fasting plasma glucose measure greater than 6.9 mmol/L.

Predictor variables

We examined potential predictor variables based on established risk factors for Type 2 diabetes and those risk factors included in existing risk scores for Type 2 diabetes related outcomes (10-12, 16, 17). Table 1 shows the predictor variables considered.

For peer review only - http://bmjopen.bmj.com/site/about/guidelines.xhtml

Table 1. Potential predictor variables

Demographic information	
Age	Ethnicity
Sex	Deprivation
Medical/family history	
Family history of diabetes	Polycystic ovary syndrome (PCOS)
Cardiovascular disease	Sleep apnoea
Schizophrenia or bipolar affective disorder	Depression
Learning disabilities	Renal/kidney disease
Gestational diabetes	
Prescribed medications	
Antihypertensives	Statins
Corticosteroids	Aspirin
Second generation “atypical” antipsychotics	
Clinical measurements	
HbA1c	Pulse rate
Body mass index (BMI)	Serum cholesterol
Systolic blood pressure	Liver function test
Diastolic blood pressure	Waist circumference
Lifestyle factors	
Smoking status	Alcohol use

Data on demographic factors, medical and family history, prescribed medications, clinical measurements, and lifestyle factors were obtained from CPRD (and HES for ethnicity). Age in single years at the index date was used. Ethnicity was derived from HES as white or non-white and when unavailable, the most recent code in CPRD was used. Deprivation was measured using the 2010 Index of Multiple Deprivation quintiles (1=least material deprivation; 5=most material deprivation). The closest value to the index date was selected for continuous measures including BMI, systolic and diastolic blood pressure, pulse rate, serum cholesterol, liver function test, and waist circumference, restricting to values recorded within six months before the index date. BMI is automatically calculated within the medical record based on input height and weight. Biologically implausible values were excluded including serum cholesterol outside of 1-15 mmol/L, systolic blood pressure outside of 20-250 mmHg, diastolic blood pressure outside of 30-150 mmHg, and BMI outside of 9-96 kg/m^2. Prescribed medications (yes or no) were determined from one or more prescription records within six months before the index date. Alcohol use (entity type=5) and smoking (entity type=4) were defined using records indicating current smoking or alcohol use within one year before the index date. All

For peer review only - http://bmjopen.bmj.com/site/about/guidelines.xhtml

others were considered non-current smokers and/or alcohol users- including former smokers and/or alcohol users. Medical and family history was determined from a diagnosis code before the index date.

Handling of missing data

Potential predictor variables with missing data for more than 33.3% of the study cohort were excluded, as these are most likely not collected as part of routine primary care (Supplementary Table S2). Assuming data were missing at random and based on previous research, multiple imputation was used to generate five imputed datasets (16, 18). Missing ethnicity (white or non-white), serum cholesterol, and systolic and diastolic blood pressure were imputed using chained equations.

Development of the models

Modelling was performed using the Stata stpm2 command for fitting flexible parametric survival models on the log cumulative hazard scale (19). Null flexible parametric models were fitted to estimate Type 2 diabetes risk using between one and five degrees of freedom to model the baseline hazard function: the final degrees of freedom was determined from visual examination of the plots of the baseline hazard functions as well as Akaike information criterion (AIC) and Bayesian information criterion (BIC) statistics. Multivariable fractional polynomial models were considered that included fractional polynomial transformations of potential continuous predictor variables. This process selects fractional polynomial models that best predict the outcome of interest. Then, manual backwards stepwise selection was used to eliminate variables that did not contribute significantly to the model using a significance threshold typical for prognostic model research of p=0.20 (20). Clinically relevant variables determined *a priori* including HbA1c, sex, and age were forced to remain in the model regardless of the p-value.

From here, two separate sex-specific models were developed. The model for females considered all of the potential predictor variables available for at least 66.6% of the study cohort. The model for

For peer review only - http://bmjopen.bmj.com/site/about/guidelines.xhtml

males did not include polycystic ovarian syndrome or gestational diabetes as potential predictor variables. The following steps were followed separately for the male and female models: 1) flexible parametric modelling was used to fit the final prognostic model and Rubin's rules were applied to combine the results across the imputed datasets; 2) the linear predictor was calculated for each patient; 3) Harrell's C statistics, Somers' D statistics, and calibration slopes were calculated for each imputed dataset and averaged (21).

Validation of the models

The models were internally validated to correct for over-fitting. Internal validation was performed separately for the male and female models. The same methodology used for multiple imputation in the development dataset was used for the validation dataset. Internal validation was performed as described by Harrell *et al.* and Snee (22, 23). The developed model was applied to the validation dataset and the performance was quantified (22). A global shrinkage factor (the mean calibration slope) was applied to the beta coefficients from the developed model. The restricted cubic splines and constant were re-estimated to maintain overall calibration (24).

Four risk groups (high, medium high, medium low, and low) were defined by the 25th, 50th and 75th percentiles of the linear predictor (the model's prognostic index distribution). A Kaplan–Meier curve was plotted for all four groups. Discrimination was visualised by the difference in observed Type 2 diabetes-free probability among the groups.

To evaluate the calibration, each imputed dataset was divided into deciles based on the linear predictor of Type 2 diabetes risk. The predicted probability of developing Type 2 diabetes (x-axis) and the observed fraction that developed Type 2 diabetes at 10 years (y-axis) were plotted for each decile risk group. The slope of this line is the calibration slope; a reference line showing perfect calibration was also plotted.

For peer review only - http://bmjopen.bmj.com/site/about/guidelines.xhtml

All analyses were performed in Stata 15 and SAS v9.4; nominal statistical significance was defined at $p<0.05$.

Patient and public involvement

Members of the public were involved in the priority-setting and question-development stages of this study.

For peer review only

RESULTS

Study population

A total of 289,754 adult patients were identified from CPRD with an HbA1c test result indicating NDH on or before December 31, 2017. Patients were excluded if they had pre-existing Type 2 diabetes (n=58,296) or Type 1 diabetes (n=822). Patients with one or more prescriptions for metformin within six months before the index date were also excluded (n=10,260). Patients were further excluded if the first recorded test indicating NDH occurred before the start of the study on January 1, 2000 (n=65,370), or if the date of death preceded the date of the first recorded test indicating NDH (n=301) as these data were likely misreported. There were 154,705 patients that met the inclusion criteria and were included in the cohort (Supplementary Figure S1); 109,077 patients were included in the development dataset (50,049 males and 59,028 females) and 45,628 patients in the validation dataset (21,014 males and 24,614 females).

In the development dataset, there were 9,332 patients, including 4,719 males and 4,613 females, diagnosed with Type 2 diabetes during a total of 293,238 person-years of follow-up. The mean follow-up for the development dataset was 2.7 years (SD 2.4, range 0-10 years). In the validation dataset, there were 3,783 patients, including 1,893 males and 1,890 females, diagnosed with Type 2 diabetes during a total of 115,113 person-years of follow-up. The mean follow-up for the validation dataset was 2.5 years (SD 2.3, range 0-10 years).

Baseline characteristics

Table 2 shows the baseline characteristics of patients in the development and validation datasets and for patients with no missing data. The distributions of continuous variables in the development and validation datasets are shown in Supplementary Figure S2.

For peer review only - http://bmjopen.bmj.com/site/about/guidelines.xhtml

Table 2. Characteristics of cohort at the index date in total, by number of missing variables, and by dataset.

			Missing variables		Dataset	
		Total	One or more	None	Development	Validation
Total		N=154,705	N=91,409	N=63,296	N=109,077	N=45,628
Age (years)		64.9 (14.2)	64.2 (14.9)	65.9 (13.1)	64.8 (14.2)	65.0 (14.2)
Sex	Male	71,063 (45.9%)	40,518 (44.3%)	30,545 (48.3%)	50,049 (45.9%)	21,014 (46.1%)
	Female	83,642 (54.1%)	50,891 (55.7%)	32,751 (51.7%)	59,028 (54.1%)	24,614 (53.9%)
Ethnicity	Non-white	14,116 (12.4%)	6,683 (13.3%)	7,433 (11.7%)	10,239 (12.9%)	3,877 (11.2%)
	White	99,468 (87.6%)	43,605 (86.7%)	55,863 (88.3%)	68,870 (87.1%)	30,598 (88.8%)
	Unknown	41,121	41,121	0	29,968	11,153
Current alcohol user		31,722 (20.5%)	14,867 (16.3%)	16,855 (26.6%)	22,320 (20.5%)	9,402 (20.6%)
Current smoker		21,126 (13.7%)	11,677 (12.8%)	9,449 (14.9%)	14,861 (13.6%)	6,265 (13.7%)
Medication	Antihypertensives	90,005 (58.2%)	47,424 (51.9%)	42,581 (67.3%)	63,290 (58.0%)	26,715 (58.5%)
	Atypical antipsychotics	3,959 (2.6%)	2,541 (2.8%)	1,418 (2.2%)	2,845 (2.6%)	1,114 (2.4%)
	Aspirin	41,986 (27.1%)	22,404 (24.5%)	19,582 (30.9%)	29,726 (27.3%)	12,260 (26.9%)
	Corticosteroids	55,090 (35.6%)	33,167 (36.3%)	21,923 (34.6%)	38,918 (35.7%)	16,172 (35.4%)
	Statins	74,166 (47.9%)	39,425 (43.1%)	34,741 (54.9%)	52,393 (48.0%)	21,773 (47.7%)
Medical/family history	Schizophrenia/bipolar	2,093 (1.4%)	1,189 (1.3%)	904 (1.4%)	1,493 (1.4%)	600 (1.3%)
	Cardiovascular disease	18,483 (11.9%)	9,608 (10.5%)	8,875 (14.0%)	12,862 (11.8%)	5,621 (12.3%)
	Depression	42,364 (27.4%)	26,066 (28.5%)	16,298 (25.7%)	29,627 (27.2%)	12,737 (27.9%)
	Learning disability	744 (0.5%)	446 (0.5%)	298 (0.5%)	478 (0.4%)	266 (0.6%)
	Diabetes in family	195 (0.1%)	117 (0.1%)	78 (0.1%)	159 (0.1%)	36 (0.1%)
	PCOS	840 (0.5%)	595 (0.7%)	245 (0.4%)	576 (0.5%)	264 (0.6%)
	Gestational diabetes	762 (0.5%)	592 (0.6%)	170 (0.3%)	567 (0.5%)	195 (0.4%)
	Renal/kidney disease	17,126 (11.1%)	9,109 (10.0%)	8,017 (12.7%)	11,810 (10.8%)	5,316 (11.7%)
	Sleep apnoea	2,289 (1.5%)	1,317 (1.4%)	972 (1.5%)	1,594 (1.5%)	695 (1.5%)
Clinical measures	HbA1c (mmol/mol)	43.5 (1.5)	43.5 (1.5)	43.5 (1.5)	43.5 (1.5)	43.5 (1.5)
	Cholesterol (mmol/L)	5.2 (1.2)	5.3 (1.2)	5.2 (1.2)	5.2 (1.2)	5.2 (1.2)
	Systolic BP (mmHg)	138.1 (18.5)	137.8 (18.8)	138.2 (18.4)	138.0 (18.6)	138.2 (18.5)
	Diastolic BP (mmHg)	80.0 (11.0)	79.6 (11.0)	80.2 (11.0)	79.9 (11.0)	80.1 (10.9)

BP=blood pressure. PCOS= Polycystic ovarian syndrome. Continuous variables are given as the mean (SD). Categorical variables are given as the number (%).Index of multiple deprivation, BMI, pulse, liver function test, and waist circumference are not included in the table since these measures are not available for >33.3% of the cohort.

For peer review only - http://bmjopen.bmj.com/site/about/guidelines.xhtml

The development dataset included 54.1% female and 12.9% non-white ethnicity; corresponding values in the validation dataset were 53.9% and 11.2%. Within the development dataset, 20.5% of patients were current alcohol users and 13.6% were current smokers compared with 20.6% and 13.7%, respectively, within the validation dataset. The percentage of patients with prescriptions of each medication was similar between the development and validation datasets. The most commonly prescribed medication was antihypertensives (58.0% in the development and 58.5% in the validation dataset), while the least common was atypical antipsychotics (2.6% and 2.4%, respectively). Of the 38,918 patients prescribed corticosteroids in the development dataset, 10,711 (27.5%) were prescribed oral medication, 19,192 were non-oral (49.3%), and 9,015 were prescribed both (23.2%; data not shown). For the validation dataset, there were 16,172 patients prescribed corticosteroids including 4,637 (28.7%) oral, 7,781 (48.1%) non-oral, and 3,754 prescribed both (23.2%). The medical/family history was similar between the development and validation datasets. The most common medical/family history condition was depression (27.2% in the development and 27.9% in the validation dataset), while the least common was a family history of diabetes (0.1% in both datasets). The mean HbA1c at the index date was the same for development and validation patients, 43.5mmol/mol (SD 1.2) or 6.1% (0.1%). Further, observed cholesterol and blood pressure were similar between the development and validation datasets.

Incidence rates of Type 2 diabetes

Supplementary Table S3 shows the incidence of Type 2 diabetes in total and in the development and validation datasets. The total incidence of Type 2 diabetes was 32.1 (95% CI 31.6-32.7) per 1,000 person-years (py): 31.8 (95% CI 31.2-32.5) in the development and 32.9 (95% CI 31.8-33.9) in the validation dataset. The largest rate difference between the development and validation datasets was for patients with a history of learning disability; the rate was 30.0 (95% CI 21.1-42.7) per 1,000 py in the development dataset compared with 41.2 (95% CI 27.6-61.5) in the validation dataset.

For peer review only - http://bmjopen.bmj.com/site/about/guidelines.xhtml

Predictor variables

Variables missing for more than 33.3% of the study cohort were eliminated as potential predictor variables including waist circumference (missing for 99.3% of patients), liver function test (99.2% missing), pulse rate (86.5% missing), BMI (73.6% missing), and deprivation (41.1% missing).

For flexible parametric modelling, three degrees of freedom were selected for the restricted cubic spline function used for the baseline hazard (AIC= 81,482, BIC= 81,520). This places two knots at percentile positions 33 and 67 of the distribution of the uncensored log survival times. Linear was the best fit for all continuous potential predictor variables; no fractional polynomial transformations were selected.

The following potential predictor variables were removed during the backwards selection process: atypical antipsychotics, cholesterol, history of a learning disability, a history of depression, a history of schizophrenia or bipolar affective disorder, and ethnicity. The final male model comprised 14 predictor variables including HbA1c, systolic blood pressure, diastolic blood pressure, age, smoking, alcohol use; prescribed medications: antihypertensives, aspirin, corticosteroids, statins; and medical history of: cardiovascular disease, renal/kidney disease, sleep apnoea; and family history of diabetes (Table 3). The female model included two additional predictors, medical history of polycystic ovarian syndrome and gestational diabetes (Table 3).

For peer review only

Table 3. Development and final coefficients for the male and female prognostic models.

Predictor	Male					Female				
	Development model				Final model	Development model				Final model
	Coefficient	95% CI		p value	Coefficient	Coefficient	95% CI		p value	Coefficient
HbA1c (mmol/mol)	0.35048	0.33231	0.36866	0.000	0.34124	0.38494	0.36673	0.40315	0.000	0.38255
Age	-0.00310	-0.00579	-0.00040	0.024	-0.00302	-0.00465	-0.00737	-0.00193	0.001	-0.00462
Current alcohol user	0.05866	-0.00659	0.12391	0.078	0.05711	0.03588	-0.03874	0.11050	0.346	0.03566
Current smoker	-0.13053	-0.21393	-0.04714	0.002	-0.12709	-0.11355	-0.20407	-0.02302	0.014	-0.11284
Antihypertensive	0.13787	-0.03490	0.31064	0.118	0.13423	0.23830	-0.01509	0.49169	0.065	0.23682
Aspirin	0.10917	0.04131	0.17703	0.002	0.10629	0.13078	0.06142	0.20015	0.000	0.12997
Corticosteroids	0.13683	0.07441	0.19926	0.000	0.13322	0.12593	0.05951	0.19234	0.000	0.12515
Statins	0.65113	0.58046	0.72180	0.000	0.63396	0.66886	0.60170	0.73603	0.000	0.66471
Cardiovascular disease	-0.08578	-0.16955	-0.00201	0.045	-0.08352	-0.11919	-0.22249	-0.01590	0.024	-0.11845
Diabetes in family	0.65379	0.10842	1.19917	0.019	0.63655	0.37641	-0.31827	1.07110	0.288	0.37408
Polycystic ovarian syndrome	-	-	-	-	-	0.22766	-0.08223	0.53755	0.150	0.22625
Gestational diabetes	-	-	-	-	-	0.49865	0.24068	0.75661	0.000	0.49555
Renal/kidney disease	-0.05138	-0.15758	0.05481	0.343	-0.05003	-0.13741	-0.23253	-0.04229	0.005	-0.13655
Sleep apnoea	0.08901	-0.09730	0.27532	0.349	0.08666	0.35832	0.04615	0.67048	0.024	0.35609
Systolic blood pressure (mmHg)	0.00594	0.00383	0.00805	0.000	0.00578	0.00599	0.00347	0.00852	0.000	0.00596
Diastolic blood pressure (mmHg)	0.00359	0.00009	0.00708	0.044	0.00349	0.00053	-0.00333	0.00439	0.784	0.00053
Restricted cubic spline 1	0.96661	0.94161	0.99160	0.000	0.96661	0.93046	0.90612	0.95481	0.000	0.93046
Restricted cubic spline 2	-0.03565	-0.05114	-0.02016	0.000	-0.03565	-0.02957	-0.04468	-0.01445	0.000	-0.02957
Restricted cubic spline 3	0.03708	0.02516	0.04901	0.000	0.03708	0.01933	0.00740	0.03127	0.002	0.01933
Constant	-19.55409	-20.40687	-18.70131	0.000	-19.55409	-20.84774	-21.70300	-19.99247	0.000	-20.84774

For peer review only - http://bmjopen.bmj.com/site/about/guidelines.xhtml

Calibration

Using the developed model, Supplementary Figure S3 shows an example of the calibration between expected and observed probabilities of developing Type 2 diabetes at 10 years of follow up within one of the imputed female and male validation datasets. There were slight differences between plots from the different imputed datasets due to the different values imputed for predictors. Using Rubin's rules to combine the results across imputed datasets, the calibration slope was 0.974 (95% CI 0.905-1.042) for males and 0.994 (95% CI 0.931-1.057) for females. This indicates that the developed models were slightly overfitted. A uniform shrinkage factor (S=0.974 for males and S=0.994 for females) was applied to each developed model's beta coefficients before recalibrating the baseline function of the final model.

Discrimination

There was relatively good separation, or discrimination, between risk groups for both males and females when the developed models were fitted using the validation dataset. Supplementary Figure S4 shows an example using one of the imputed validation datasets. There were slight differences between plots from the different imputed datasets due to the different values imputed for predictors. For both males and females, the log-rank test for all imputed datasets indicated that the survivor functions were different between risk groups ($p<0.001$ for both males and females). Furthermore, validation showed that the male model discriminated reasonably well with mean Harrell's C statistic across imputed datasets of 0.701 and Somers' D statistic of 0.402; for the female model, the corresponding statistics were 0.718 and 0.436 (Table 4). These values suggest slightly better discrimination for the female model.

For peer review only - http://bmjopen.bmj.com/site/about/guidelines.xhtml

Table 4. Male and female prognostic model mean performance statistics across imputed datasets.

Measure	Male		Female	
	Development	Validation	Development	Validation
Harrell's C	0.700	0.701	0.720	0.718
Somers' D	0.401	0.402	0.441	0.436
Calibration slope	1.000	0.974	1.000	0.994

For peer review only

For peer review only - http://bmjopen.bmj.com/site/about/guidelines.xhtml

DISCUSSION

Although several prognostic and diagnostic models for predicting Type 2 diabetes-related outcomes have been developed and validated within the UK, none to date has been specifically developed in a population with NDH, for whom the risk profile is likely different than the general population. The available evidence shows that the incidence of Type 2 diabetes in the cohort of patients used to develop the QDiabetes-2018 risk assessment tool was 4.17 (95% CI 4.15 to 4.19) per 1,000 person-years (16). Those included in our study were significantly more likely to develop Type 2 diabetes. In fact, the incidence in our development cohort was nearly eight times that of the QDiabetes-2018 development cohort. Therefore, we have developed and validated pragmatic sex-specific prognostic models for predicting the risk of developing Type 2 diabetes in those with NDH, which could be used for targeting referral to the NHS DPP. Our models include important risk factors for people that already have NDH.

Since the primary aim of this study was to develop models that could be easily implemented using routinely collected data, in the variable selection process we closely considered data availability and excluded variables with high levels of missing data, including waist circumference, liver function, pulse rate, BMI, and deprivation. Waist circumference and BMI are key risk factors for Type 2 diabetes, but these measures may not be obtained due to lack of time and other practical or perceived barriers (24). BMI, in particular, has been included in many existing Type 2 diabetes models (10). However, the inclusion of BMI must be balanced with practicality, given that our data showed BMI (or height and weight) were infrequently recorded in a primary care setting.

Since the models were developed using observational primary care data, the accuracy of coding, particularly of the outcome, has the potential to affect model development. Research published in 2011 found that miscoding, misdiagnosis, and misclassification of diabetes was common in UK primary care (25). However, in more recent years, implementation of the UK Quality and Outcomes Framework

For peer review only - http://bmjopen.bmj.com/site/about/guidelines.xhtml

(QoF) has resulted in better coding of Type 2 diabetes, specifically within CPRD (26, 27). With improved interoperability, the launch of SNOMED is expected to further boost coding accuracy (28). Since this research utilised data initially recorded for managing the care of individual patients, there are also a number of potential sources of bias. To address this, the study cohort included only patients that are considered by CPRD of acceptable research standards. Further, clinical measures that were not biologically plausible and likely misreported were excluded. In most cases, another value that was biologically plausible was available within the same period for the patient.

This study has several strengths. These models are for use in primary care. Therefore, we used a primary care database (CPRD) to develop the models. In recent years the HbA1c assay has been the preferred method to diagnose NDH and Type 2 diabetes compared with oral glucose tolerance or fasting plasma glucose tests (29). Therefore, these models were developed using HbA1c to quantify blood glucose. The large sample size allowed for a sufficient number of events per predictor parameter. We considered a range of predictors specifically selected due to clinical relevance to development of Type 2 diabetes. Continuous predictors were not categorised, so there was no loss of information. The decision to develop sex-specific models was based on the presence of some sex-specific risk factors, like history of gestational diabetes. Additionally, we identified new risk factors not included in the 2018 update of QDiabetes, which was developed within the general population (16). These risk factors include history of sleep apnoea, blood pressure, alcohol use, prescription of antihypertensives, and prescription of aspirin.

This study also had several limitations. The primary limitation is the splitting of the cohort into development and validation datasets instead of using a fully external database to validate the model. However, given the size of the cohort and the large number of events, this likely had little effect on model development. Furthermore, to ensure case mix, non-random selection was used to split the cohort. The outcome for this study was defined using a single medcode or test result indicating Type

For peer review only - http://bmjopen.bmj.com/site/about/guidelines.xhtml

2 diabetes. In practice, this would typically be confirmed via a follow up test. Another limitation is that the models included predictor variables obtained at one point in time including a single HbA1c measure to determine NDH. However, the models could be adjusted to include time-varying predictors relatively easily. Methods such as land marking or joint models could be used to model changes in predictors over time. Some predictor variables were self-reported including smoking, alcohol use, and family history of diabetes. The proportion of non-current smokers is in line with a similar study while the proportion of patients with a family history of diabetes in this study was much lower than that reported in a similar study.(16) This may indicate that family history of diabetes is not established in clinical practice or established but not recorded within the CPRD. Prescriptions issued were used as a proxy for current medication. Patients may not have filled the prescription or adhered to the medication. Because this was an open cohort and the number of people diagnosed with NDH has increased in recent years, the mean follow-up time was short- 2.7 years for patients in the development dataset and 2.5 years for patients in the validation dataset. However, 14,896 patients in the development dataset and 5,678 patients in the validation dataset had five or more years of follow up. Therefore, based on existing research, we believe that there was sufficient follow-up time to determine risk for progression to Type 2 diabetes. HES and ONS linkage was only available for 59.0% of patients in the cohort. If linkage to ONS was not available and a date of death was provided in CPRD, then the CPRD date was used. While ONS is the gold standard for date of death, deaths are less well coded in CPRD. It is possible that deaths for some patients without linkage to ONS were never coded in CPRD, and the patients were not censored accordingly. However, this likely only affected a few patients. Finally, there may have been additional predictor variables that were not considered either because they are not collected as part of routine clinical care or because they are not among the known traditional risk factors for Type 2 diabetes.

Similar to the QRISK cardiovascular disease risk algorithm, the models presented are designed to be integrated into primary care computer systems to automatically calculate risk (30). At the time of the

For peer review only - http://bmjopen.bmj.com/site/about/guidelines.xhtml

For peer review only

first HbA1c test indicating NDH, a risk score could be automatically generated using the HbA1c measure along with clinical, prescription, and diagnoses data already contained in the individual's electronic health record. Additionally, the algorithm for imputing missing data could also be implemented automatically. Rather than referring all adults with NDH to the NHS DPP, healthcare providers could prioritize referrals for people at high risk for progressing to Type 2 diabetes.

The NHS DPP is a limited resource and does not have current capacity to accommodate all adults with NDH in England. People are referred to the NHS DPP through the NHS Health Check programme, aimed at people aged 40-74, or people with NDH identified through opportunistic assessment or as part of routine clinical care (9). Eligibility for the NHS DPP is typically determined through an HbA1c measure or, less frequently, an Oral Glucose Tolerance Test (OGTT). However, this study has identified additional factors to stratify further the risk of developing Type 2 diabetes within this high-risk group. Targeting referrals may be a more cost-effective and efficient way to deliver the NHS DPP. The male and female prognostic models we developed and validated could be used to identify and target those most at risk of developing Type 2 diabetes for referral to the NHS DPP. Implementation of these models would standardise the NHS DPP identification and referral process to be consistent across sites and based on information already collected as part of primary care. The next step is to determine the optimum risk threshold to accurately identify patients that will develop Type 2 diabetes.

For peer review only

For peer review only - http://bmjopen.bmj.com/site/about/guidelines.xhtml

Footnotes

CRediT Author Statement

Conceptualization: LJG, FZ, MJD, KK; funding acquisition: LJG (lead), MJD, FZ; methodology, writing – original draft: BC, LJG; data curation, formal analysis, validation, visualization: BC; software: BC, SB; writing – review & editing: FZ, MJD, KK, SB. All authors provided final approval of the version to publish. The corresponding author (BC) had full access to all the data in the study and had final responsibility for the decision to submit it for publication.

Competing interests

BC, LJG, FZ, and SB: none

MJD has acted as consultant, advisory board member and speaker for Novo Nordisk, Sanofi-Aventis, Lilly, Merck Sharp & Dohme, Boehringer Ingelheim, AstraZeneca and Janssen, an advisory board member for Servier and as a speaker for Mitsubishi Tanabe Pharma Corporation and Takeda Pharmaceuticals International Inc. She has received grants in support of investigator and investigator initiated trials from Novo Nordisk, Sanofi-Aventis, Lilly, Boehringer Ingelheim and Janssen. She was a member of the NICE public health guideline for prevention of Type 2 diabetes (NICE PH 38).

KK has acted as a consultant and speaker for Novartis, Novo Nordisk, Sanofi-Aventis, Lilly and Merck Sharp & Dohme. He has received grants in support of investigator and investigator-initiated trials from Novartis, Novo Nordisk, Sanofi-Aventis, Lilly, Pfizer, Boehringer Ingelheim and Merck Sharp & Dohme. He is a member of the External Reference Group of the NHS DPP and was Chair of the NICE public health guideline for prevention of Type 2 diabetes (NICE PH 38).

Acknowledgements

This study is based on data from the Clinical Practice Research Datalink GOLD database obtained under licence from the UK Medicines and Healthcare products Regulatory Agency. However, the interpretation and conclusions contained in this article are those of the authors alone.

The authors gratefully acknowledge Leicester Real-World Evidence Unit (LRWE) for facilitating the download of CPRD data. LRWE is funded by University of Leicester, National Institute for Health Research (NIHR) Applied Research Collaboration East Midlands (ARC EM) and NIHR Leicester Biomedical Research Centre. The interpretation and conclusions contained in this report/article do not necessarily reflect those of the LRWE.

Funding

This research was supported by a grant from National Institute for Health Research (NIHR) Collaboration for Leadership in Applied Health Research and Care (CLAHRC) East Midlands. The funding body had no role in the study design, data collection, analysis, or interpretation; in the writing of the manuscript; or in the decision to submit the manuscript.

For peer review only - http://bmjopen.bmj.com/site/about/guidelines.xhtml

For peer review only

This research was also funded in part by the NIHR Leicester Biomedical Research Centre and NIHR Applied Research Collaboration. The views expressed are those of the author(s) and not necessarily those of the NHS, the NIHR or the Department of Health and Social Care.

Ethical approval

This research was approved by the Independent Scientific Advisory Committee (ISAC) for Medicines and Healthcare products Regulatory Agency Database Research (protocol 18_238).

Data sharing

Patient-level electronic health records obtained from CPRD cannot be shared. However, the authors will share programming code and aggregate statistics if requested. A list of medcodes used to define Type 2 diabetes, pre-existing Type 1 diabetes, and medical and family history as well as product codes used to identify current medication is available at https://github.com/bc188/Prognostic-model-codes.

For peer review only

REFERENCES

1. Barron E, Valabhji J, Young B. Prevalence, characteristics, distribution and identification of non-diabetic hyperglycaemia in England, 2016. Available from https://wwwgovuk/government/news/five-million-people-at-high-risk-of-type-2-diabetes. Accessed 20 Sept 2019
2. Gillies CL, Abrams KR, Lambert PC, Cooper NJ, Sutton AJ, Hsu RT, Khunti K. Pharmacological and lifestyle interventions to prevent or delay type 2 diabetes in people with impaired glucose tolerance: systematic review and meta-analysis. BMJ 2007;334:299
3. Kramer MK, Miller RG, Siminerio LM Evaluation of a community Diabetes Prevention Program delivered by diabetes educators in the United States: One-year follow up. Diabetes Res Clin Pr 2014;106(3):E49-E52.
4. Knowler WC B-CE, Fowler SE, et al. Reduction in the incidence of type 2 diabetes with lifestyle intervention or metformin. N Engl J Med 2002; 346:393-403
5. Tuomilehto J, Lindström J, Eriksson JG, Valle TT, Hämäläinen H, Ilanne-Parikka P, Keinänen-Kiukaanniemi S, Laakso M, Louheranta A, Rastas M, Salminen V, Uusitupa M. Prevention of Type 2 Diabetes Mellitus by Changes in Lifestyle among Subjects with Impaired Glucose Tolerance. N Engl J Med 2001; 344:1343-1350
6. Pan XR, Li GW, Hu YH, Wang JX, Yang WY, An ZX, Hu ZX, Lin J, Xiao JZ, Cao HB, Liu PA, Jiang XG, Jiang YY, Wang JP, Zheng H, Zhang H, Bennett PH, Howard BV. Effects of diet and exercise in preventing NIDDM in people with impaired glucose tolerance: the Da Qing IGT and Diabetes Study. Diabetes Care 1997; 20:537-544
7. Troughton J, Chatterjee S, Hill SE, Daly H, Martin Stacey L, Stone MA, Patel N, Khunti K, Yates T, Gray LJ, Davies MJ. Development of a lifestyle intervention using the MRC framework for diabetes prevention in people with impaired glucose regulation. J Public Health (Oxf) 38: 493-501
8. England NHS. Roll out of the Programme, 2016. Available from https://www.england.nhs.uk/diabetes/diabetes-prevention/roll-out-of-the-programme. Accessed 20 Sept 2019
9. England NHS. NHSDPP overview and FAQ, 2016. Available from https://www.england.nhs.uk/wp-content/uploads/2016/08/dpp-faq.pdf. Accessed 20 Sept 2019
10. Collins GS, Mallett S, Omar O, Yu L. Developing risk prediction models for type 2 diabetes: a systematic review of methodology and reporting. BMC Med 2011;9:103
11. Noble D, Mathur R, Dent T, Meads C, Greenhalgh T. Risk models and scores for type 2 diabetes: systematic review. BMJ 2011;343(d7163).
12. Barber SR, Davies MJ, Khunti K, Gray LJ. Risk assessment tools for detecting those with pre-diabetes: a systematic review. Diabetes Res Clin Pract 2014;105:1-13
13. Bodicoat DH, Khunti K, Srinivasan BT, Mostafa S, Gray LJ, Davies MJ, Webb DR. Incident Type 2 diabetes and the effect of early regression to normoglycaemia in a population with impaired glucose regulation. Diabet Med 2017;34:396-404
14. Herrett E, Gallagher AM, Bhaskaran K, Forbes H, Mathur R, van Staa T, Smeeth L. Data resource profile: clinical practice research datalink (CPRD). Int J Epidemiol 2015;44:827-36
15. Riley RD, Snell KI, Ensor J, Burke DL, Harrell Jr FE, Moons KG, Collins GS. Minimum sample size for developing a multivariable prediction model: PART II-binary and time-to-event outcomes. Stat Med 2019;38:1276-96
16. Hippisley-Cox J, Coupland C. Development and validation of QDiabetes-2018 risk prediction algorithm to estimate future risk of type 2 diabetes: cohort study. BMJ 2017;359:j5019
17. Gray LJ, Taub N, Khunti K, Gardiner E, Hiles S, Webb DR, Srinivasan BT, Davies MJ. The Leicester Risk Assessment score for detecting undiagnosed Type 2 diabetes and impaired glucose regulation for use in a multiethnic UK setting. Diabet Med 2010;27:887-95
18. Van der Heijden GJ, Donders ART, Stijnen T, Moons KG. Imputation of missing values is superior to complete case analysis and the missing-indicator method in multivariable diagnostic research: a clinical example. J Clin Epidemiol 2006;59:1102-9
19. Royston P, Lambert PC. Flexible parametric survival analysis using Stata: beyond the Cox model. Stat Med 2014;33:5280-97
20. Dunkler D, Plischke M, Leffondré K, Heinze G. Augmented backward elimination: a pragmatic and purposeful way to develop statistical models. PLoS One 2014;9:e113677
21. Harrell FE, Califf RM, Pryor DB, Lee KL, Rosati RA. Evaluating the yield of medical tests. JAMA 1982;247:2543-6

For peer review only - http://bmjopen.bmj.com/site/about/guidelines.xhtml

22. Harrell FE, Lee KL, Mark DB. Multivariable prognostic models: Issues in developing models, evaluating assumptions and adequacy, and measuring and reducing errors. Stat Med 1996;15:361-87
23. Snee R. Validation of Regression Models: Methods and Examples. Technometrics 1977;19:419-28
24. Dunkley AJ, Stone MA, Patel N, Davies MJ, Khunti K. Waist circumference measurement: knowledge, attitudes and barriers in patients and practitioners in a multi-ethnic population. Fam Pract 2009;26:365-71
25. De Lusignan S, Sadek N, Mulnier H, Tahir A, Russell-Jones D, Khunti K. Miscoding, misclassification and misdiagnosis of diabetes in primary care. Diabet Med 2012;29:181-9
26. Calvert M, Shankar A, McManus RJ, Lester H, Freemantle N. Effect of the quality and outcomes framework on diabetes care in the United Kingdom: retrospective cohort study. BMJ 2009;338:b1870
27. Tate AR, Dungey S, Glew S, Beloff N, Williams R, Williams T. Quality of recording of diabetes in the UK: how does the GP's method of coding clinical data affect incidence estimates? Cross-sectional study using the CPRD database. BMJ open 2017;7:e012905
28. Haarbrandt B, Schreiweis B, Rey S, Sax U, Scheithauer S, Rienhoff O, Knaup-Gregori P, Bavendiek U, Dieterich C, Brors B, Kraus I, Thoms CM, Jäger D, Ellenrieder V, Bergh B, Yahyapour R, Eils R, Consortium H, Marschollek M. HiGHmed - An Open Platform Approach to Enhance Care and Research across Institutional Boundaries. Methods Inf Med 2018;57:e66-e81
29. International Expert Committee. International Expert Committee report on the role of the A1C assay in the diagnosis of diabetes. Diabetes Care 2009;32:1327-34
30. Hippisley-Cox J, Coupland C, Brindle P. Development and validation of QRISK3 risk prediction algorithms to estimate future risk of cardiovascular disease: prospective cohort study. BMJ. 2017;357:j2099

For peer review only

Supplementary Table S1. Number of practices by region in total and included in the development and validation datasets.

Practice region	Total	Dataset	
		Development	Validation
North East	11	8	3
North West	85	60	26
Yorkshire & The Humber	28	20	8
East Midlands	25	18	8
West Midlands	61	43	18
East of England	54	38	16
South West	61	43	18
South Central	56	39	17
London	95	67	29
South East Coast	68	48	20
Northern Ireland	25	18	8
Scotland	94	66	28
Wales	77	54	23
**Total**	**740**	**518**	**222**

For peer review only

Supplementary Table S2. Percent of patients missing potential predictor variables.

Predictor variable	Missing	
	n	%
Waist circumference	153,592	99.3
Liver function test	153,493	99.2
Pulse rate	133,890	86.5
BMI	113,840	73.6
Index of Multiple Deprivation	63,524	41.1
Systolic blood pressure	48,390	31.3
Diastolic blood pressure	48,390	31.3
Ethnicity	41,121	26.6
Serum cholesterol	38,910	25.2
HbA1c	0	0
Age	0	0
Sex	0	0
Current alcohol use	0	0
Current smoker	0	0
Antihypertensives	0	0
Atypical antipsychotics	0	0
Aspirin	0	0
Corticosteroids	0	0
Statins	0	0
Bipolar disease or schizophrenia	0	0
Cardiovascular disease	0	0
Depression	0	0
Learning disability	0	0
Diabetes in family	0	0
Polycystic ovarian syndrome	0	0
Gestational diabetes	0	0
Renal/kidney disease	0	0
Sleep apnoea	0	0

For peer review only

Supplementary Table S3. Incidence of Type 2 diabetes per 1,000 person years with 95% confidence intervals.

		Total			Dataset					
					Development			Validation		
		Py	n	Rate (95% CI)	Py	n	Rate (95% CI)	Py	n	Rate (95% CI)
Total		408,350.5	13,115	32.1 (31.6-32.7)	293,237.8	9,332	31.8 (31.2-32.5)	115,112.6	3,783	32.9 (31.8-33.9)
Age group	<30	4,285.1	79	18.4 (14.8-23.0)	3,017.0	56	18.6 (14.3-24.1)	1,268.1	23	18.1 (12.1-27.3)
	30-39	15,214.7	307	20.2 (18.0-22.6)	11,050.8	231	20.9 (18.4-23.8)	4,164.0	76	18.3 (14.6-22.9)
	40-49	43,354.3	1,157	26.7 (25.2-28.3)	31,539.3	836	26.5 (24.8-28.4)	11,815.0	321	27.2 (24.4-30.3)
	50-59	81,437.4	2,399	29.5 (28.3-30.7)	58,691.3	1,730	29.5 (28.1-30.9)	22,746.1	669	29.4 (27.3-31.7)
	60-69	109,599.6	3,808	34.7 (33.7-35.9)	79,177.3	2,709	34.2 (32.9-35.5)	30,422.4	1,099	36.1 (34.1-38.3)
	70-79	96,100.4	3,553	37.0 (35.8-38.2)	68,493.3	2,527	36.9 (35.5-38.4)	27,607.1	1,026	37.2 (35.0-39.5)
	80-89	50,818.9	1,629	32.1 (30.5-33.7)	36,072.2	1,114	30.9 (29.1-32.8)	14,746.7	515	34.9 (32.0-38.1)
	90+	7,540.0	183	24.3 (21.0-28.1)	5,196.7	129	24.8 (20.9-29.5)	2,343.2	54	23.0 (17.6-30.1)
Sex	Male	186,953.5	6,612	35.4 (34.5-36.2)	134,390.2	4,719	35.1 (34.1-36.1)	52,563.3	1,893	36.0 (34.4-37.7)
	Female	221,397.0	6,503	29.4 (28.7-30.1)	158,847.6	4,613	29.0 (28.2-29.9)	62,549.3	1,890	30.2 (28.9-31.6)
Ethnicity	Non-white	38,606.0	1,154	29.9 (28.2-31.7)	29,281.3	863	29.5 (27.6-31.5)	9,324.7	291	31.2 (27.8-35.0)
	White	257,231.3	8,446	32.8 (32.1-33.5)	181,622.3	5,878	32.4 (31.5-33.2)	75,609.0	2,568	34.0 (32.7-35.3)
Current alcohol user	No	321,672.8	10,049	31.2 (30.6-31.9)	231,489.6	7,223	31.2 (30.5-31.9)	90,183.2	2,826	31.3 (30.2-32.5)
	Yes	86,677.6	3,066	35.4 (34.1-36.6)	61,748.2	2,109	34.2 (32.7-35.6)	24,929.4	957	38.4 (36.0-40.9)
Current smoker	No	351,866.5	11,355	32.3 (31.7-32.9)	252,907.8	8,103	32.0 (31.3-32.7)	98,958.7	3,252	32.9 (31.8-34.0)
	Yes	56,483.9	1,760	31.2 (29.7-32.6)	40,330.0	1,229	30.5 (28.8-32.2)	16,154.0	531	32.9 (30.2-35.8)
Antihypertensives	No	402,244.5	12,840	31.9 (31.4-32.5)	288,800.1	9,137	31.6 (31.0-32.3)	113,444.4	3,703	32.6 (31.6-33.7)
	Yes	6,105.9	275	45.0 (40.0-50.7)	4,437.7	195	43.9 (38.2-50.6)	1,668.3	80	48.0 (38.5-59.7)
Atypical antipsychotics	No	397,003.1	12,760	32.1 (31.6-32.7)	284,987.3	9,084	31.9 (31.2-32.5)	112,015.8	3,676	32.8 (31.8-33.9)
	Yes	11,347.4	355	31.3 (28.2-34.7)	8,250.5	248	30.1 (26.5-34.0)	3,096.9	107	34.6 (28.6-41.8)
Aspirin	No	282,265.5	7,971	28.2 (27.6-28.9)	202,397.8	5,686	28.1 (27.4-28.8)	79,867.7	2,285	28.6 (27.5-29.8)
	Yes	126,085.0	5,144	40.8 (39.7-41.9)	90,840.0	3,646	40.1 (38.9-41.5)	35,245.0	1,498	42.5 (40.4-44.7)
Corticosteroids	No	132,237.8	3,781	28.6 (27.7-29.5)	94,557.1	2,721	28.8 (27.7-29.9)	37,680.7	1,060	28.1 (26.5-29.9)
	Yes	276,112.7	9,334	33.8 (33.1-34.5)	198,680.8	6,611	33.3 (32.5-34.1)	77,431.9	2,723	35.2 (33.9-36.5)
Statins	No	197,618.7	4,184	21.2 (20.5-21.8)	141,932.3	2,977	21.0 (20.2-21.7)	55,686.3	1,207	21.7 (20.5-22.9)
	Yes	210,731.8	8,931	42.4 (41.5-43.3)	151,305.5	6,355	42.0 (41.0-43.0)	59,426.3	2,576	43.3 (41.7-45.1)
Schizophrenia/bipolar	No	402,889.4	12,937	32.1 (31.6-32.7)	289,246.4	9,212	31.8 (31.2-32.5)	113,642.9	3,725	32.8 (31.7-33.8)
	Yes	5,461.1	178	32.6 (28.1-37.8)	3,991.4	120	30.1 (25.1-36.0)	1,469.7	58	39.5 (30.5-51.0)
Cardiovascular disease	No	361,574.5	11,297	31.2 (30.7-31.8)	260,237.0	8,074	31.0 (30.4-31.7)	101,337.5	3,223	31.8 (30.7-32.9)
	Yes	46,776.0	1,818	38.9 (37.1-40.7)	33,000.8	1,258	38.1 (36.1-40.3)	13,775.1	560	40.7 (37.4-44.2)
Depression	No	303,786.2	9,875	32.5 (31.9-33.2)	219,040.2	7,043	32.2 (31.4-32.9)	84,746.0	2,832	33.4 (32.2-34.7)

For peer review only

	Yes	104,564.3	3,240	31.0 (29.9-32.1)	74,197.7	2,289	30.9 (29.6-32.1)	30,366.6	951	31.3 (29.4-33.4)
Learning disability	No	406,734.9	13,060	32.1 (31.6-32.7)	292,204.8	9,301	31.8 (31.2-32.5)	114,530.1	3,759	32.8 (31.8-33.9)
	Yes	1,615.6	55	34.0 (26.1-44.3)	1,033.0	31	30.0 (21.1-42.7)	582.5	24	41.2 (27.6-61.5)
Diabetes in family	No	407,867.0	13,091	32.1 (31.6-32.7)	292,821.8	9,311	31.8 (31.2-32.4)	115,045.1	3,780	32.9 (31.8-33.9)
	Yes	483.5	24	49.6 (33.3-74.1)	416.0	21	50.5 (32.9-77.4)	67.5	<5	44.4 (14.3-137.8)
Renal/kidney disease	No	368,309.2	11,766	31.9 (31.4-32.5)	265,292.2	8,403	31.7 (31.0-32.4)	103,016.9	3,363	32.6 (31.6-33.8)
	Yes	40,041.3	1,349	33.7 (31.9-35.5)	27,945.6	929	33.2 (31.2-35.5)	12,095.7	420	34.7 (31.6-38.2)
Sleep apnoea	No	403,300.5	12,896	32.0 (31.4-32.5)	289,628.0	9,178	31.7 (31.0-32.3)	113,672.4	3,718	32.7 (31.7-33.8)
	Yes	5,050.0	219	43.4 (38.0-49.5)	3,609.8	154	42.7 (36.4-50.0)	1,440.2	65	45.1 (35.4-57.6)
PCOS*	No	219,461.5	6,441	29.3 (28.6-30.1)	157,488.6	4,571	29.0 (28.2-29.9)	61,972.9	1,870	30.2 (28.8-31.6)
	Yes	1,935.5	62	32.0 (25.0-41.1)	1,359.0	42	30.9 (22.8-41.8)	576.5	20	34.7 (22.4-53.8)
Gestational diabetes*	No	219,205.1	6,423	29.3 (28.6-30.0)	157,163.0	4,550	29.0 (28.1-29.8)	62,042.1	1,873	30.2 (28.9-31.6)
	Yes	2,191.9	80	36.5 (29.3-45.4)	1,684.7	63	37.4 (29.2-47.9)	507.2	17	33.5 (20.8-53.9)
HbA1c (mmol/mol)	42	143,564.4	2,341	16.3 (15.7-17.0)	102,303.4	1,650	16.1 (15.4-16.9)	41,261.0	691	16.7 (15.5-18.0)
	43	103,706.7	2,496	24.1 (23.1-25.0)	74,289.4	1,762	23.7 (22.6-24.9)	29,417.3	734	25.0 (23.2-26.8)
	44	72,839.5	2,563	35.2 (33.9-36.6)	52,495.5	1,801	34.3 (32.8-35.9)	20,344.0	762	37.5 (34.9-40.2)
	45	48,523.8	2,407	49.6 (47.7-51.6)	35,497.9	1,724	48.6 (46.3-50.9)	13,025.9	683	52.4 (48.6-56.5)
	46	31,687.9	2,473	78.0 (75.0-81.2)	22,985.0	1,794	78.1 (74.5-81.7)	8,702.9	679	78.0 (72.4-84.1)
	47	8,028.2	835	104.0 (97.2-111.3)	5,666.6	601	106.1 (97.9-114.9)	2,361.6	234	99.1 (87.2-112.6)
Cholesterol (mmol/L)	<5.0	130,946.3	4,568	34.9 (33.9-35.9)	93,611.8	3,273	35.0 (33.8-36.2)	37,334.5	1,295	34.7 (32.8-36.6)
	5.0-6.9	152,342.9	4,978	32.7 (31.8-33.6)	109,313.4	3,548	32.5 (31.4-33.5)	43,029.5	1,430	33.2 (31.6-35.0)
	≥7.0	24,848.2	817	32.9 (30.7-35.2)	17,982.7	573	31.9 (29.4-34.6)	6,865.5	244	35.5 (31.3-40.3)
Systolic BP (mmHg)	<140	147,766.9	4,476	30.3 (29.4-31.2)	105,813.8	3,195	30.2 (29.2-31.3)	41,953.1	1,281	30.5 (28.9-32.3)
	≥140	135,710.1	5,206	38.4 (37.3-39.4)	97,560.5	3,685	37.8 (36.6-39.0)	38,149.6	1,521	39.9 (37.9-41.9)
Diastolic BP (mmHg)	<90	228,884.2	7,540	32.9 (32.2-33.7)	164,236.7	5,367	32.7 (31.8-33.6)	64,647.6	2,173	33.6 (32.2-35.1)
	≥90	54,592.8	2,142	39.2 (37.6-40.9)	39,137.6	1,513	38.7 (36.8-40.7)	15,455.2	629	40.7 (37.6-44.0)

BP=blood pressure. PCOS=Polycystic ovarian syndrome.

Table includes observed values only. Imputed values for ethnicity, serum cholesterol, and systolic and diastolic blood pressure are not included.

Index of multiple deprivation, BMI, pulse, liver function test, and waist circumference are not included in the table since these measures are not available for >33.3% of the cohort.

Age was collapsed into 10-year groups. HbA1c was collapsed into one mmol/mol increments. Cholesterol was collapsed into three clinically relevant groups (<5.0, 5.0-6.9, and ≥7.0mmol/L). Systolic blood pressure was collapsed into two clinically relevant groups based on NICE guidelines for hypertension (<140 and ≥140 mmHg) as was diastolic blood pressure (<90 and ≥90 mmHg) (27).

For peer review only

*The incidence was calculated among females only.

[+]Note, n<5 cannot be published.

For peer review only

Supplementary Figure S1. Participant flow diagram.

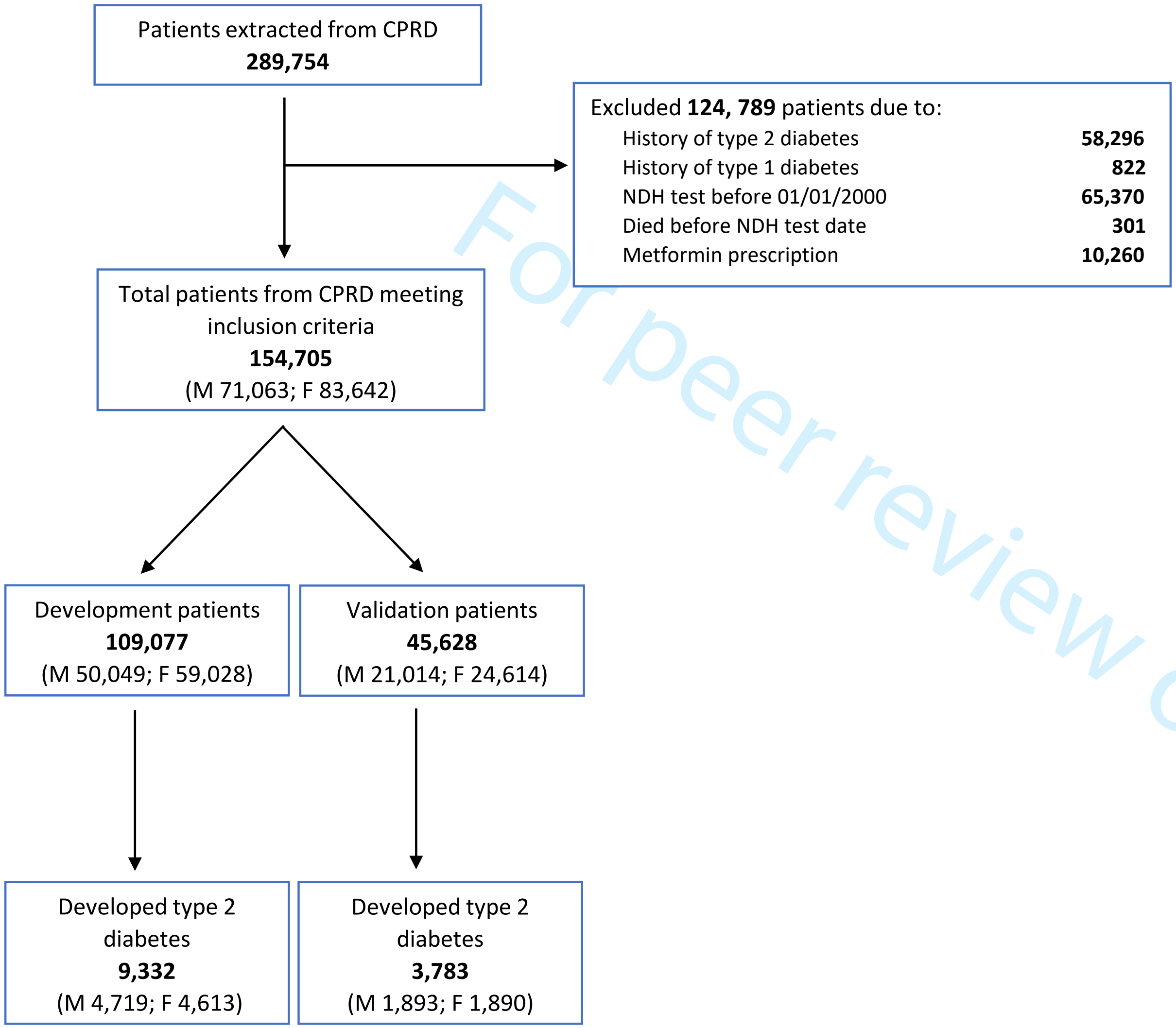

For peer review only

Supplementary Figure S2. Distribution of continuous variables for the development and validation datasets.

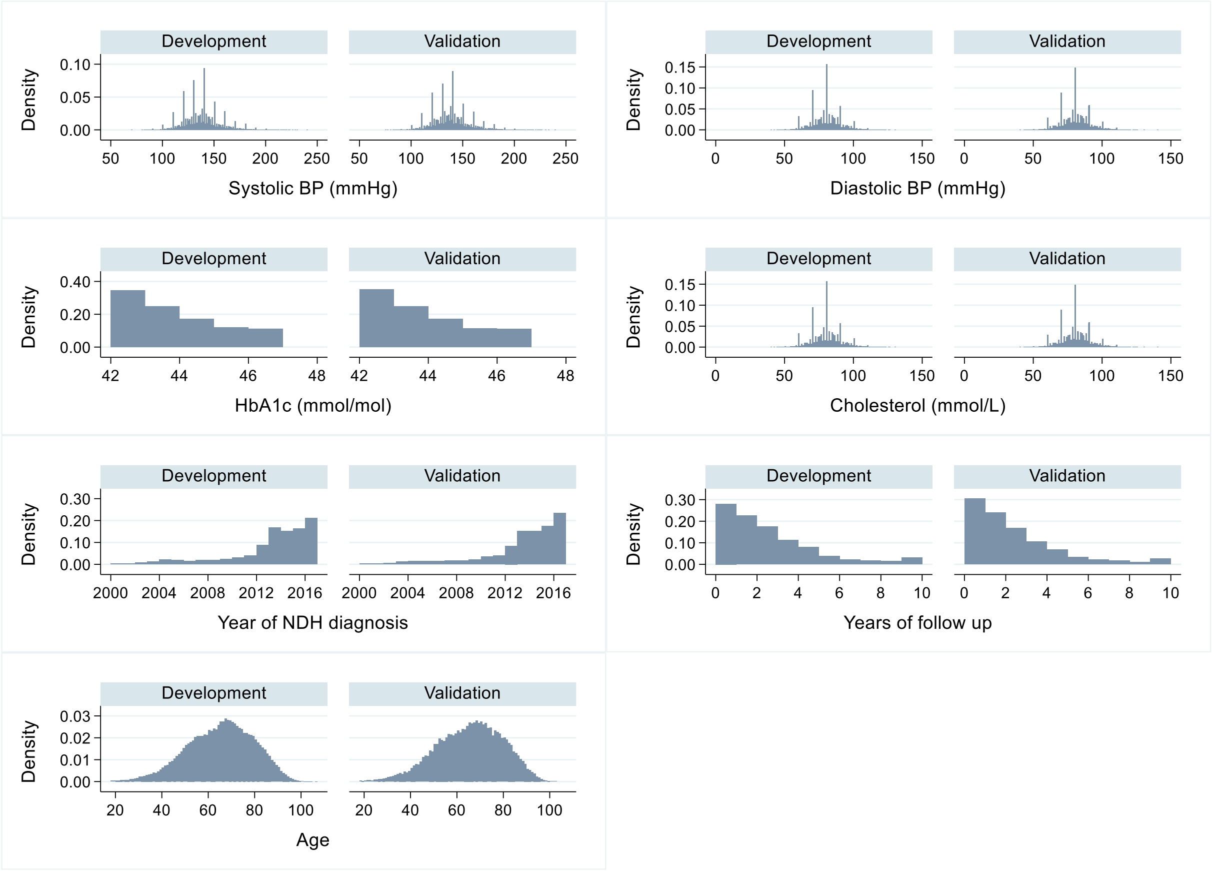

Supplementary Figure S3. Calibration plots by 10-year Type 2 diabetes risk deciles for the male and female models in one of the imputed validation datasets.

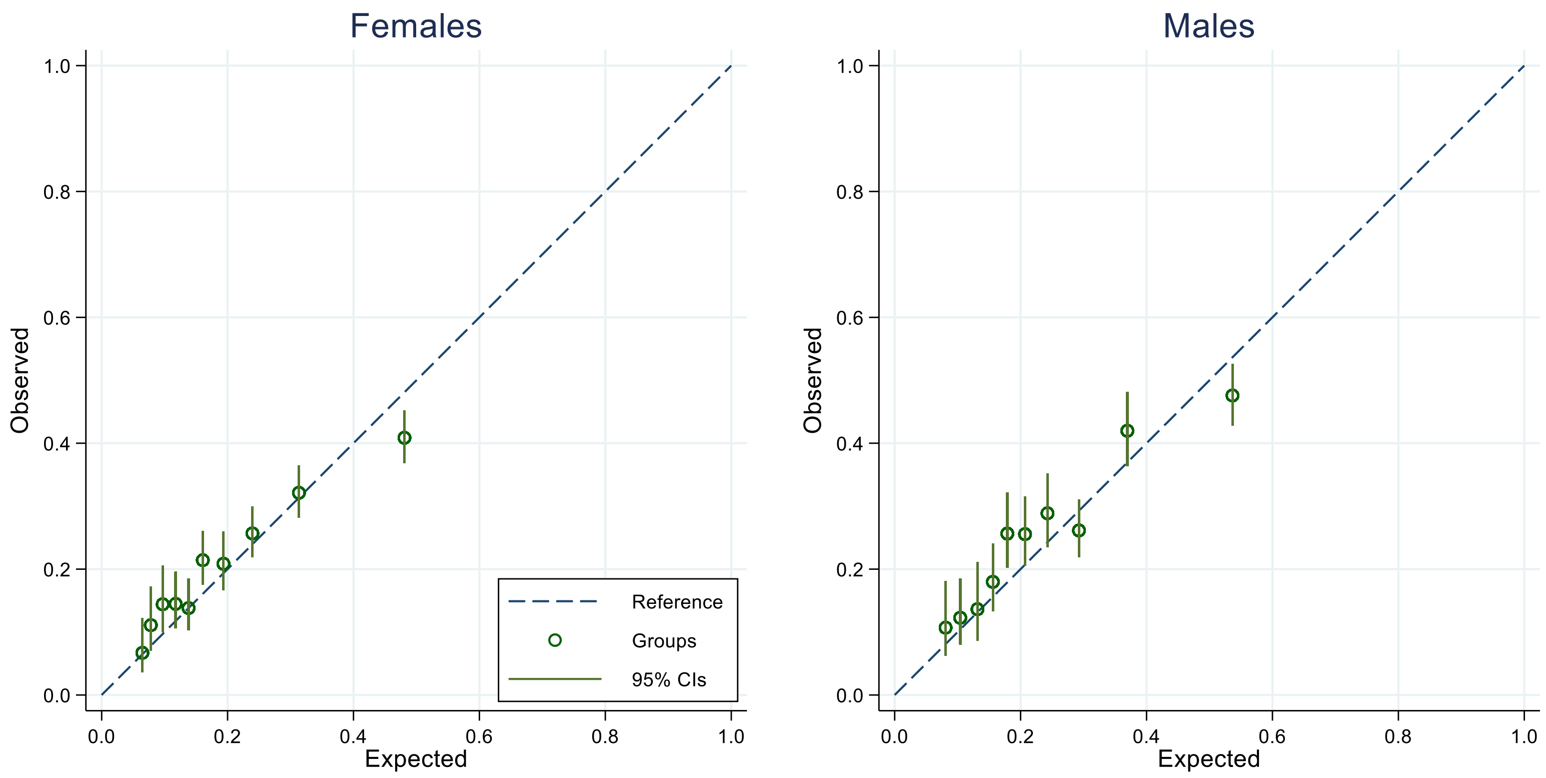

Supplementary Figure S4. Kaplan-Meier Type 2 diabetes-free probability and 95% confidence intervals for the male and female models in one of the imputed validation datasets.

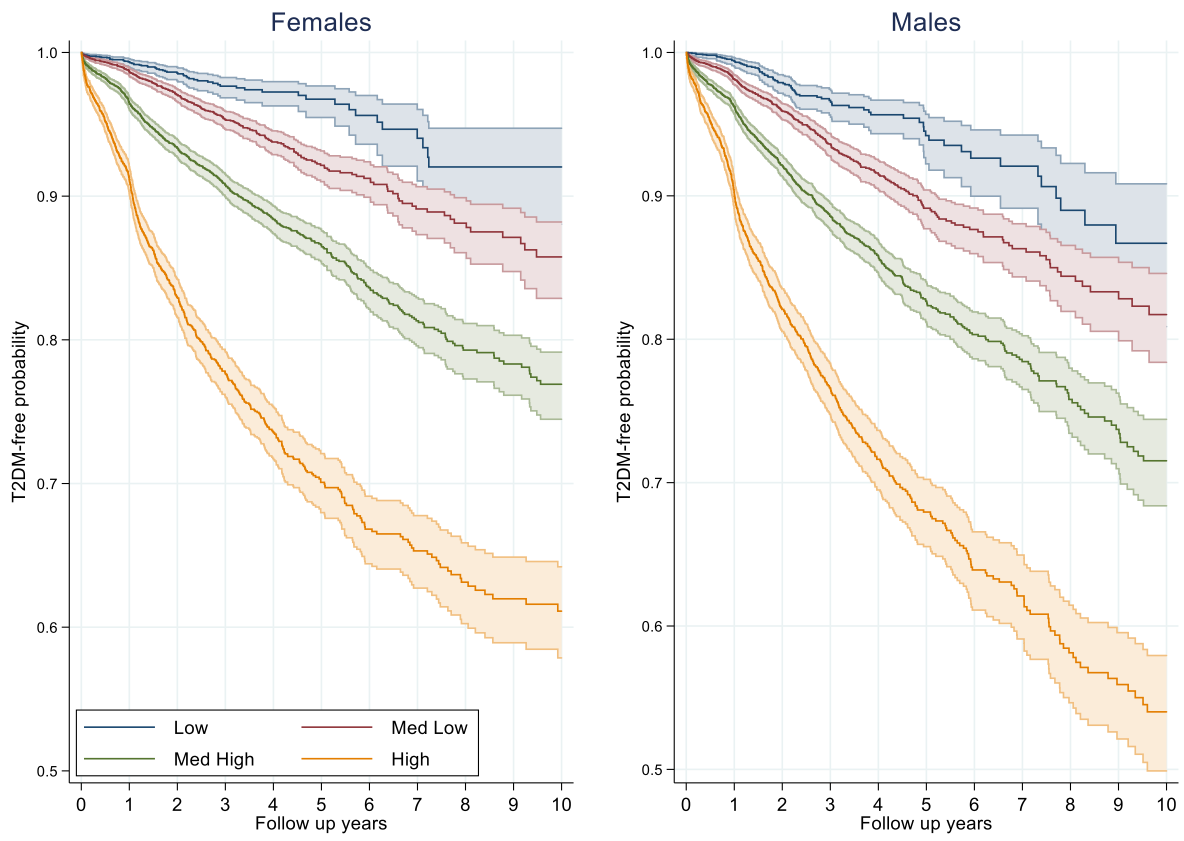

The RECORD statement – checklist of items, extended from the STROBE statement, that should be reported in observational studies using routinely collected health data.

	Item No.	STROBE items	Location in manuscript where items are reported	RECORD items	Location in manuscript where items are reported
**Title and abstract**					
	1	(a) Indicate the study's design with a commonly used term in the title or the abstract (b) Provide in the abstract an informative and balanced summary of what was done and what was found		RECORD 1.1: The type of data used should be specified in the title or abstract. When possible, the name of the databases used should be included.<br><br>RECORD 1.2: If applicable, the geographic region and timeframe within which the study took place should be reported in the title or abstract.<br><br>RECORD 1.3: If linkage between databases was conducted for the study, this should be clearly stated in the title or abstract.	Pg 1-2<br><br>Pg 2<br><br>NA
**Introduction**					
Background rationale	2	Explain the scientific background and rationale for the investigation being reported			Pg 4-5
Objectives	3	State specific objectives, including any prespecified hypotheses			Pg 5
**Methods**					
Study Design	4	Present key elements of study design early in the paper			Pg 6
Setting	5	Describe the setting, locations, and relevant dates, including periods of recruitment, exposure, follow-up, and data collection			Pg 6-7

For peer review only

Participants	6	*(a) Cohort study* - Give the eligibility criteria, and the sources and methods of selection of participants. Describe methods of follow-up<br>*Case-control study* - Give the eligibility criteria, and the sources and methods of case ascertainment and control selection. Give the rationale for the choice of cases and controls<br>*Cross-sectional study* - Give the eligibility criteria, and the sources and methods of selection of participants<br><br>*(b) Cohort study* - For matched studies, give matching criteria and number of exposed and unexposed<br>*Case-control study* - For matched studies, give matching criteria and the number of controls per case		RECORD 6.1: The methods of study population selection (such as codes or algorithms used to identify subjects) should be listed in detail. If this is not possible, an explanation should be provided.<br><br>RECORD 6.2: Any validation studies of the codes or algorithms used to select the population should be referenced. If validation was conducted for this study and not published elsewhere, detailed methods and results should be provided.<br><br>RECORD 6.3: If the study involved linkage of databases, consider use of a flow diagram or other graphical display to demonstrate the data linkage process, including the number of individuals with linked data at each stage.	Pg 6-8, link to code lists provided on Pg 22<br><br>NA<br><br>Pg 6
Variables	7	Clearly define all outcomes, exposures, predictors, potential confounders, and effect modifiers. Give diagnostic criteria, if applicable.		RECORD 7.1: A complete list of codes and algorithms used to classify exposures, outcomes, confounders, and effect modifiers should be provided. If these cannot be reported, an explanation should be provided.	link to code lists provided on Pg 22
Data sources/ measurement	8	For each variable of interest, give sources of data and details of methods of assessment (measurement).<br>Describe comparability of assessment methods if there is more than one group			Pg 6-8

For peer review only

Bias	9	Describe any efforts to address potential sources of bias			Pg 18-19
Study size	10	Explain how the study size was arrived at			Pg 7
Quantitative variables	11	Explain how quantitative variables were handled in the analyses. If applicable, describe which groupings were chosen, and why			NA
Statistical methods	12	(a) Describe all statistical methods, including those used to control for confounding<br>(b) Describe any methods used to examine subgroups and interactions<br>(c) Explain how missing data were addressed<br>(d) *Cohort study* - If applicable, explain how loss to follow-up was addressed<br>*Case-control study* - If applicable, explain how matching of cases and controls was addressed<br>*Cross-sectional study* - If applicable, describe analytical methods taking account of sampling strategy<br>(e) Describe any sensitivity analyses			Pg 6-11
Data access and cleaning methods		..		RECORD 12.1: Authors should describe the extent to which the investigators had access to the database population used to create the study population.	Pg 6, Figure S1

For peer review only

				RECORD 12.2: Authors should provide information on the data cleaning methods used in the study.	Pg 7
Linkage		..		RECORD 12.3: State whether the study included person-level, institutional-level, or other data linkage across two or more databases. The methods of linkage and methods of linkage quality evaluation should be provided.	Pg 6<br>Linkage was not performed by the research team, rather linked data are obtained from CPRD directly
**Results**					
Participants	13	(a) Report the numbers of individuals at each stage of the study (*e.g.*, numbers potentially eligible, examined for eligibility, confirmed eligible, included in the study, completing follow-up, and analysed)<br>(b) Give reasons for non-participation at each stage.<br>(c) Consider use of a flow diagram		RECORD 13.1: Describe in detail the selection of the persons included in the study (*i.e.,* study population selection) including filtering based on data quality, data availability and linkage. The selection of included persons can be described in the text and/or by means of the study flow diagram.	Pg 5
Descriptive data	14	(a) Give characteristics of study participants (*e.g.*, demographic, clinical, social) and information on exposures and potential confounders<br>(b) Indicate the number of participants with missing data for each variable of interest<br>(c) *Cohort study* - summarise follow-up time (*e.g.*, average and total amount)			Table 2
Outcome data	15	*Cohort study* - Report numbers of outcome events or summary measures over time<br>*Case-control study* - Report numbers in each exposure			Figure S1

For peer review only

		category, or summary measures of exposure<br>*Cross-sectional study* - Report numbers of outcome events or summary measures			
Main results	16	(a) Give unadjusted estimates and, if applicable, confounder-adjusted estimates and their precision (e.g., 95% confidence interval). Make clear which confounders were adjusted for and why they were included<br>(b) Report category boundaries when continuous variables were categorized<br>(c) If relevant, consider translating estimates of relative risk into absolute risk for a meaningful time period			Table 3<br><br>Supp. Table S3 caption<br><br>NA
Other analyses	17	Report other analyses done—e.g., analyses of subgroups and interactions, and sensitivity analyses			NA
**Discussion**					
Key results	18	Summarise key results with reference to study objectives			Pg 12-16
Limitations	19	Discuss limitations of the study, taking into account sources of potential bias or imprecision. Discuss both direction and magnitude of any potential bias		RECORD 19.1: Discuss the implications of using data that were not created or collected to answer the specific research question(s). Include discussion of misclassification bias, unmeasured confounding, missing data, and changing eligibility over time, as they pertain to the study being reported.	Pg 13
Interpretation	20	Give a cautious overall interpretation of results considering objectives,			Pg 17-20

For peer review only

		limitations, multiplicity of analyses, results from similar studies, and other relevant evidence			
Generalisability	21	Discuss the generalisability (external validity) of the study results			Pg 20
**Other Information**					
Funding	22	Give the source of funding and the role of the funders for the present study and, if applicable, for the original study on which the present article is based			Pg 21
Accessibility of protocol, raw data, and programming code		..		RECORD 22.1: Authors should provide information on how to access any supplemental information such as the study protocol, raw data, or programming code.	Pg 22

*Reference: Benchimol EI, Smeeth L, Guttmann A, Harron K, Moher D, Petersen I, Sørensen HT, von Elm E, Langan SM, the RECORD Working Committee. The REporting of studies Conducted using Observational Routinely-collected health Data (RECORD) Statement. *PLoS Medicine* 2015; in press.

*Checklist is protected under Creative Commons Attribution (CC BY) license.

For peer review only

TRIPOD Checklist: Prediction Model Development and Validation

Section/Topic	Item		Checklist Item	Page
**Title and abstract**				
Title	1	D;V	Identify the study as developing and/or validating a multivariable prediction model, the target population, and the outcome to be predicted.	**1**
Abstract	2	D;V	Provide a summary of objectives, study design, setting, participants, sample size, predictors, outcome, statistical analysis, results, and conclusions.	**2-3**
**Introduction**				
Background and objectives	3a	D;V	Explain the medical context (including whether diagnostic or prognostic) and rationale for developing or validating the multivariable prediction model, including references to existing models.	**4-5**
	3b	D;V	Specify the objectives, including whether the study describes the development or validation of the model or both.	**5**
**Methods**				
Source of data	4a	D;V	Describe the study design or source of data (e.g., randomized trial, cohort, or registry data), separately for the development and validation data sets, if applicable.	**6**
	4b	D;V	Specify the key study dates, including start of accrual; end of accrual; and, if applicable, end of follow-up.	**6**
Participants	5a	D;V	Specify key elements of the study setting (e.g., primary care, secondary care, general population) including number and location of centres.	**6**
	5b	D;V	Describe eligibility criteria for participants.	**6**
	5c	D;V	Give details of treatments received, if relevant.	**NA**
Outcome	6a	D;V	Clearly define the outcome that is predicted by the prediction model, including how and when assessed.	**7-8**
	6b	D;V	Report any actions to blind assessment of the outcome to be predicted.	**NA**
Predictors	7a	D;V	Clearly define all predictors used in developing or validating the multivariable prediction model, including how and when they were measured.	**8-9**
	7b	D;V	Report any actions to blind assessment of predictors for the outcome and other predictors.	**NA**
Sample size	8	D;V	Explain how the study size was arrived at.	**7**
Missing data	9	D;V	Describe how missing data were handled (e.g., complete-case analysis, single imputation, multiple imputation) with details of any imputation method.	**9**
Statistical analysis methods	10a	D	Describe how predictors were handled in the analyses.	**8-9**
	10b	D	Specify type of model, all model-building procedures (including any predictor selection), and method for internal validation.	**9-10**
	10c	V	For validation, describe how the predictions were calculated.	**10-11**
	10d	D;V	Specify all measures used to assess model performance and, if relevant, to compare multiple models.	**11**
	10e	V	Describe any model updating (e.g., recalibration) arising from the validation, if done.	**11**
Risk groups	11	D;V	Provide details on how risk groups were created, if done.	**11**
Development vs. validation	12	V	For validation, identify any differences from the development data in setting, eligibility criteria, outcome, and predictors.	**10**
**Results**				
Participants	13a	D;V	Describe the flow of participants through the study, including the number of participants with and without the outcome and, if applicable, a summary of the follow-up time. A diagram may be helpful.	**Fig S1**
	13b	D;V	Describe the characteristics of the participants (basic demographics, clinical features, available predictors), including the number of participants with missing data for predictors and outcome.	**Tab 2**
	13c	V	For validation, show a comparison with the development data of the distribution of important variables (demographics, predictors and outcome).	**Sup Fig S2**
Model development	14a	D	Specify the number of participants and outcome events in each analysis.	**Fig S1**
	14b	D	If done, report the unadjusted association between each candidate predictor and outcome.	**NA**
Model specification	15a	D	Present the full prediction model to allow predictions for individuals (i.e., all regression coefficients, and model intercept or baseline survival at a given time point).	**Tab 3**
	15b	D	Explain how to the use the prediction model.	**13**
Model performance	16	D;V	Report performance measures (with CIs) for the prediction model.	**Tab 4**
Model-updating	17	V	If done, report the results from any model updating (i.e., model specification, model performance).	**19-20**
**Discussion**				
Limitations	18	D;V	Discuss any limitations of the study (such as nonrepresentative sample, few events per predictor, missing data).	**13**
Interpretation	19a	V	For validation, discuss the results with reference to performance in the development data, and any other validation data.	**Tab 4**
	19b	D;V	Give an overall interpretation of the results, considering objectives, limitations, results from similar studies, and other relevant evidence.	**18-19**
Implications	20	D;V	Discuss the potential clinical use of the model and implications for future research.	**20**
**Other information**				
Supplementary information	21	D;V	Provide information about the availability of supplementary resources, such as study protocol, Web calculator, and data sets.	**22**
Funding	22	D;V	Give the source of funding and the role of the funders for the present study.	**21**

*Items relevant only to the development of a prediction model are denoted by D, items relating solely to a validation of a prediction model are denoted by V, and items relating to both are denoted D;V. We recommend using the TRIPOD Checklist in conjunction with the TRIPOD Explanation and Elaboration document.

For peer review only - http://bmjopen.bmj.com/site/about/guidelines.xhtml

BMJ Open

Prediction of Type 2 diabetes risk in people with non-diabetic hyperglycaemia: model derivation and validation using UK primary care data

Journal:	*BMJ Open*
Manuscript ID	bmjopen-2020-037937.R1
Article Type:	Original research
Date Submitted by the Author:	29-Jul-2020
Complete List of Authors:	Coles, Briana; University of Leicester,<br>Khunti, Kamlesh; University of Leicester, Department of Health Sciences<br>Booth, Sarah; University of Leicester, Department of Health Sciences<br>Zaccardi, Francesco; University of Leicester, Diabetes Research Centre<br>Davies, Melanie; University of Leicester, Diabetes Research Centre<br>Gray, Laura; University of Leicester, Department of Health Sciences
**Primary Subject Heading**:	Diabetes and endocrinology
Secondary Subject Heading:	Patient-centred medicine, General practice / Family practice, Epidemiology, Evidence based practice, Diagnostics
Keywords:	General diabetes < DIABETES & ENDOCRINOLOGY, PRIMARY CARE, EPIDEMIOLOGY, Health policy < HEALTH SERVICES ADMINISTRATION & MANAGEMENT, Risk management < HEALTH SERVICES ADMINISTRATION & MANAGEMENT, Diabetes & endocrinology < INTERNAL MEDICINE

SCHOLARONE™
Manuscripts

I, the Submitting Author has the right to grant and does grant on behalf of all authors of the Work (as defined in the below author licence), an exclusive licence and/or a non-exclusive licence for contributions from authors who are: i) UK Crown employees; ii) where BMJ has agreed a CC-BY licence shall apply, and/or iii) in accordance with the terms applicable for US Federal Government officers or employees acting as part of their official duties; on a worldwide, perpetual, irrevocable, royalty-free basis to BMJ Publishing Group Ltd ("BMJ") its licensees and where the relevant Journal is co-owned by BMJ to the co-owners of the Journal, to publish the Work in this journal and any other BMJ products and to exploit all rights, as set out in our licence.

The Submitting Author accepts and understands that any supply made under these terms is made by BMJ to the Submitting Author unless you are acting as an employee on behalf of your employer or a postgraduate student of an affiliated institution which is paying any applicable article publishing charge ("APC") for Open Access articles. Where the Submitting Author wishes to make the Work available on an Open Access basis (and intends to pay the relevant APC), the terms of reuse of such Open Access shall be governed by a Creative Commons licence – details of these licences and which Creative Commons licence will apply to this Work are set out in our licence referred to above.

Other than as permitted in any relevant BMJ Author's Self Archiving Policies, I confirm this Work has not been accepted for publication elsewhere, is not being considered for publication elsewhere and does not duplicate material already published. I confirm all authors consent to publication of this Work and authorise the granting of this licence.

For peer review only

Prediction of Type 2 diabetes risk in people with non-diabetic hyperglycaemia: model derivation and validation using UK primary care data

Short running title

Type 2 diabetes risk prediction in people with non-diabetic hyperglycaemia

Authors

Briana Coles[1,3], Kamlesh Khunti[1,3], Sarah Booth[2], Francesco Zaccardi [1,3], Melanie J Davies[3], Laura J Gray[2]

Affiliations

[1]Leicester Real World Evidence Unit, Diabetes Research Centre, University of Leicester, Leicester, UK.

[2]Department of Health Sciences, University of Leicester, Leicester, UK.

[3]Diabetes Research Centre, University of Leicester, Leicester General Hospital, Leicester, UK.

Corresponding author

Briana Coles, bc188@leicester.ac.uk

Gwendolen Rd, Leicester LE5 4PW, UK

Tel: +44 (0) 734 213 7177

Email: bc188@le.ac.uk

Word count

Abstract: 284

Main Text: 4,466

Tables 4; RECORD checklist; TRIPOD checklist; ISAC application; Supplementary Tables 4; Supplementary Figures 4

Keywords

Type 2 Diabetes Mellitus; Prediabetic State; Hyperglycaemia; Preventive Medicine; Preventive Health Services; Evidence-Based Practice; Risk prediction; biostatistics

For peer review only - http://bmjopen.bmj.com/site/about/guidelines.xhtml

For peer review only

ABSTRACT

Objective: Using primary care data, develop and validate sex-specific prognostic models that estimate the ten year risk of people with non-diabetic hyperglycaemia developing Type 2 diabetes.

Design: Retrospective cohort study

Setting: Primary care

Participants: 154,705 adult patients with non-diabetic hyperglycaemia

Primary outcome: Development of type 2 diabetes

Methods: This study used data routinely collected in UK primary care from general practices contributing to the Clinical Practice Research Datalink. Patients were split into development (n=109,077) and validation datasets (n=45,628). Potential predictor variables- including demographic and lifestyle factors, medical and family history, prescribed medications, and clinical measures- were included in survival models following the imputation of missing data. Measures of calibration at 10 years and discrimination were determined using the validation dataset.

Results: In the development dataset, 9,332 patients developed Type 2 diabetes during 293,238 person-years of follow-up (31.8 [95% CI 31.2-32.5] per 1,000 person-years). In the validation dataset, 3,783 patients developed Type 2 diabetes during 115,113 person-years of follow-up (32.9 [95% CI 31.8-33.9] per 1,000 person-years). The final prognostic models comprised 14 and 16 predictor variables for males and females, respectively. Both models had good calibration and high levels of discrimination. The performance statistics for the male model were: Harrell's C statistic of 0.700 in the development and 0.701 in the validation dataset, with a calibration slope of 0.974 (95% CI 0.905-1.042) in the validation dataset. For the female model, Harrell's C statistics were 0.720 and 0.718, respectively, while the calibration slope was 0.994 (95% CI 0.931-1.057) in the validation dataset.

For peer review only - http://bmjopen.bmj.com/site/about/guidelines.xhtml

Conclusion: These models could be used in primary care to identify those with non-diabetic hyperglycaemia most at risk of developing Type 2 diabetes for targeted referral to the National Health Service Diabetes Prevention Programme.

For peer review only

For peer review only - http://bmjopen.bmj.com/site/about/guidelines.xhtml

STRENGTHS AND LIMITATIONS

Strengths

- A large, representative primary care database was used to develop the models using HbA1c to quantify blood glucose.
- A range of predictors were considered specifically selected due to clinical relevance to development of Type 2 diabetes.

Limitations

- The cohort was split into development and validation datasets instead of using a fully external database to validate the model, but given the size of the cohort and the large number of events, this likely had little effect on model development.
- The outcome for this study was defined using a single medcode or test result indicating Type 2 diabetes.

For peer review only

For peer review only - http://bmjopen.bmj.com/site/about/guidelines.xhtml

INTRODUCTION

People with blood glucose levels raised beyond normal but not high enough for a formal diagnosis of Type 2 diabetes (i.e. HbA1c 6.0-6.4% or 42-47 mmol/mol) are at high risk of eventually developing Type 2 diabetes. This high risk state has been termed non-diabetic hyperglycaemia (NDH) or prediabetes (1). In 2015 in England it was estimated that there were five million people aged 16 years and over with NDH, a prevalence of 11.4% (1). The prevalence was much lower in people younger than 40 years of age, with the exception of minority ethnic populations (1). Evidence from large-scale clinical trials has shown that the development of Type 2 diabetes can be delayed or even prevented if those with NDH are enrolled into a diabetes prevention programme (2, 3).

Diabetes prevention programmes encourage participants to change their behaviour with a focus on increasing physical activity, improving diet quality and reducing weight. These programmes have been developed and tested internationally (2, 4-6). Initially studies focused on very intensive programmes – for example a programme developed and tested within the US involved 16 one to one individualised sessions over six months, followed by monthly individual and group based sessions to reinforce messages (4). Over a mean follow-up of 2.8 years, there was a 58% reduction (95% CI 48%-66%) in the risk of Type 2 diabetes in those randomised to receive the prevention programme compared to standard care (4). Other studies conducted in Finland and China with similar programmes found comparable results (5, 6). Such resource intensive programmes, although very effective, are not viable for delivery within an NHS setting.

Therefore, emphasis shifted to developing a more pragmatic programme that could be delivered in a group setting and requires less contact time. The National Health Service's Diabetes Prevention Programme (NHS DPP) launched in 2016 and is open to adults with NDH (7, 8). The NHS estimates that once the NHS DPP is fully rolled out in 2020, 100,000 people will access the programme each year (9). Based on this, it will take over 50 years for all those with NDH to access the programme.

For peer review only - http://bmjopen.bmj.com/site/about/guidelines.xhtml

Many prognostic and diagnostic models have been developed and validated for identifying those with undiagnosed Type 2 diabetes, NDH or those at risk of developing Type 2 diabetes (10-12). Evidence shows that the risk of developing Type 2 diabetes in those with NDH is variable. Some people with NDH will revert to normal glucose levels over time, with only a subset going on to develop Type 2 diabetes (13). Indeed referring all patients with NDH to the DPP is overtreatment in the majority of cases (14). Therefore, in the era of big data and personalised medicine, utilising data stored in primary care to target referrals to those at highest risk may be a more efficient use of the NHS DPP than the current blanket referral approach.

To date no validated risk assessments for use in those with NDH have been developed for use in the UK. Therefore, we developed and validated sex-specific prognostic models to quantify the 10-year risk of those with NDH developing Type 2 diabetes using data routinely collected in primary care. Such models should be used to target referrals to the NHS DPP.

For peer review only

METHODS

Study design and data source

This observational retrospective cohort study included a sample of primary care patients from the UK who were registered with practices contributing to the Clinical Practice Research Datalink (CPRD). The CPRD includes anonymised primary care electronic health records for over 11.3 million patients from 674 UK practices dating back to 1987 (15). The CPRD includes data for approximately 6.9% of the UK population and is broadly representative of the age, sex and ethnicity of the UK general population (15). When available, patients were also linked to Office of National Statistics (ONS) to obtain the date of death and Hospital Episode Statistics (HES) to obtain ethnicity (both available for 59% of patients in the study cohort). Linked Index of Multiple Deprivation data (quintiles) were also obtained. Approval by the CPRD Independent Scientific Advisory Committee was granted for this study (approved protocol number 18_238).

This study included an open cohort of patients registered in CPRD aged 18 years or older with NDH. NDH was defined as an HbA1c measure within 42-47 mmol/mol (6.0-6.4%). For each patient, the index date was defined as the first recorded test measurement indicating NDH between January 1, 2000 and December 31, 2017. Patients with a diagnosis of Type 2 or Type 1 diabetes before the index date were excluded. Patients with an HbA1c measure greater than 47 mmol/mol (6.4%), random blood glucose measure greater than 11.0 mmol/L (199 mg/dL), or fasting plasma glucose measure greater than 6.9 mmol/L before the index date were also excluded as these patients were assumed to be in the process of confirming a diagnosis of Type 2 diabetes. Patients prescribed metformin, the current first line therapy for Type 2 diabetes, were also excluded. Patients were followed up for a maximum of 10 years until diagnosis of Type 2 diabetes, or censoring (transferring out of practice, death, or the end of study on December 31, 2017, whichever came first).

For peer review only - http://bmjopen.bmj.com/site/about/guidelines.xhtml

The cohort was split into a development and validation dataset. To split the cohort, practices of registration were stratified by region and patients were clustered by practice (Supplementary Table S1). Approximately 33% of practices in each region were randomly assigned to the validation dataset.

Sample size

There were 71,063 males and 83,642 females meeting the inclusion criteria (Supplementary Figure S1). This resulted in 50,049 males and 59,028 females in the development dataset and 21,014 males and 24,614 females in the validation dataset. Within the development dataset, 4,719 males and 4,613 females developed Type 2 diabetes. Riley *et al.* have proposed an approach for calculating the minimum number of events per predictor parameter for a survival model based on the model's anticipated R squared, event rate, follow up time and number of predictor parameters (16). We used the R squared, event rate, and mean follow up for men and women from a similar study to estimate the required sample size.(17) For women, based on 31 predictor parameters (deprivation has five categories) considered for our study, the required minimum sample size was 3,406. For men, based on 29 predictor parameters considered for our study, the required minimum sample size was 2,585.

Outcome

The outcome was the first diagnosis of Type 2 diabetes recorded within the CPRD between January 1, 2000 and December 31, 2017. The first diagnosis of Type 2 diabetes was identified by medcode; HbA1c measure greater than 47 mmol/mol (6.4%); random blood glucose measure greater than 11.0 mmol/L (199 mg/dL); or fasting plasma glucose measure greater than 6.9 mmol/L.

Predictor variables

We examined potential predictor variables based on established risk factors for Type 2 diabetes and those risk factors included in existing risk scores for Type 2 diabetes related outcomes (10-12, 17, 18). Table 1 shows the predictor variables considered.

For peer review only - http://bmjopen.bmj.com/site/about/guidelines.xhtml

Table 1. Potential predictor variables

Demographic information	
Age	Ethnicity
Sex	Deprivation
Medical/family history	
Family history of diabetes	Polycystic ovary syndrome (PCOS)
Cardiovascular disease	Sleep apnoea
Schizophrenia or bipolar affective disorder	Depression
Learning disabilities	Renal/kidney disease
Gestational diabetes	
Prescribed medications	
Antihypertensives	Statins
Corticosteroids	Aspirin
Second generation “atypical” antipsychotics	
Clinical measurements	
HbA1c	Pulse rate
Body mass index (BMI)	Serum cholesterol
Systolic blood pressure	Liver function test
Diastolic blood pressure	Waist circumference
Lifestyle factors	
Smoking status	Alcohol use

Data on demographic factors, medical and family history, prescribed medications, clinical measurements, and lifestyle factors were obtained from CPRD (and HES for ethnicity). Age in single years at the index date was used. Ethnicity was derived from HES as white or non-white and when unavailable, the most recent code in CPRD was used. Deprivation was measured using the 2010 Index of Multiple Deprivation quintiles (1=least material deprivation; 5=most material deprivation). The closest value to the index date was selected for continuous measures including BMI, systolic and diastolic blood pressure, pulse rate, serum cholesterol, liver function test, and waist circumference, restricting to values recorded within six months before the index date. BMI is automatically calculated within the medical record based on input height and weight. Biologically implausible values were excluded including serum cholesterol outside of 1-15 mmol/L, systolic blood pressure outside of 20-250 mmHg, diastolic blood pressure outside of 30-150 mmHg, and BMI outside of 9-96 kg/m^2. Prescribed medications (yes or no) were determined from one or more prescription records within six months before the index date. Alcohol use (entity type=5) and smoking (entity type=4) were defined using records indicating current smoking or alcohol use within one year before the index date. All

For peer review only - http://bmjopen.bmj.com/site/about/guidelines.xhtml

others were considered non-current smokers and/or alcohol users- including former smokers and/or alcohol users. Medical and family history was determined from a diagnosis code before the index date.

Handling of missing data

Potential predictor variables with missing data for more than 33.3% of the study cohort were excluded, as these are most likely not collected as part of routine primary care (Supplementary Table S2). Assuming data were missing at random and based on previous research, multiple imputation was used to generate five imputed datasets (17, 19). Missing ethnicity (white or non-white), serum cholesterol, and systolic and diastolic blood pressure were imputed using chained equations.

Development of the models

Modelling was performed using the Stata stpm2 command for fitting flexible parametric survival models on the log cumulative hazard scale (20). Null flexible parametric models were fitted to estimate Type 2 diabetes risk using between one and five degrees of freedom to model the baseline hazard function: the final degrees of freedom was determined from visual examination of the plots of the baseline hazard functions as well as Akaike information criterion (AIC) and Bayesian information criterion (BIC) statistics. Multivariable fractional polynomial models were considered that included fractional polynomial transformations of potential continuous predictor variables. This process selects fractional polynomial models that best predict the outcome of interest. Then, manual backwards stepwise selection was used to eliminate variables that did not contribute significantly to the model using a significance threshold typical for prognostic model research of p=0.20 (21). Clinically relevant variables determined *a priori* including HbA1c, sex, and age were forced to remain in the model regardless of the p-value.

From here, two separate sex-specific models were developed. The model for females considered all of the potential predictor variables available for at least 66.6% of the study cohort. The model for

For peer review only - http://bmjopen.bmj.com/site/about/guidelines.xhtml

For peer review only

males did not include polycystic ovarian syndrome or gestational diabetes as potential predictor variables. The following steps were followed separately for the male and female models: 1) flexible parametric modelling was used to fit the final prognostic model and Rubin's rules were applied to combine the results across the imputed datasets; 2) the linear predictor was calculated for each patient; 3) Harrell's C statistics, Somers' D statistics, and calibration slopes were calculated for each imputed dataset and averaged (22).

Validation of the models

The models were internally validated to correct for over-fitting. Internal validation was performed separately for the male and female models. The same methodology used for multiple imputation in the development dataset was used for the validation dataset. Internal validation was performed as described by Harrell *et al.* and Snee (23, 24). The developed model was applied to the validation dataset and the performance was quantified (23). A global shrinkage factor (the mean calibration slope) was applied to the beta coefficients from the developed model. The restricted cubic splines and constant relating to the baseline of the model were re-estimated to maintain overall calibration (25).

Four risk groups (high, medium high, medium low, and low) were defined by the 15th, 50th and 85th percentiles of the linear predictor (the model's prognostic index distribution). A Kaplan–Meier curve was plotted for all four groups. Discrimination was visualised by the difference in observed Type 2 diabetes-free probability among the groups.

To evaluate the calibration, each imputed dataset was divided into deciles based on the linear predictor of Type 2 diabetes risk. The predicted probability of developing Type 2 diabetes (x-axis) and the observed fraction that developed Type 2 diabetes at 10 years (y-axis) were plotted for each decile risk group. The slope of this line is the calibration slope; a reference line showing perfect calibration was also plotted.

For peer review only - http://bmjopen.bmj.com/site/about/guidelines.xhtml

All analyses were performed in Stata 15 and SAS v9.4; nominal statistical significance was defined at $p<0.05$.

Patient and public involvement

Members of the public were involved in the priority-setting and question-development stages of this study.

For peer review only

For peer review only - http://bmjopen.bmj.com/site/about/guidelines.xhtml

RESULTS

Study population

A total of 289,754 adult patients were identified from CPRD with an HbA1c test result indicating NDH on or before December 31, 2017. Patients were excluded if they had pre-existing Type 2 diabetes (n=58,296) or Type 1 diabetes (n=822). Patients with one or more prescriptions for metformin within six months before the index date were also excluded (n=10,260). Patients were further excluded if the first recorded test indicating NDH occurred before the start of the study on January 1, 2000 (n=65,370), or if the date of death preceded the date of the first recorded test indicating NDH (n=301) as these data were likely misreported. There were 154,705 patients that met the inclusion criteria and were included in the cohort (Supplementary Figure S1); 109,077 patients were included in the development dataset (50,049 males and 59,028 females) and 45,628 patients in the validation dataset (21,014 males and 24,614 females).

In the development dataset, there were 9,332 patients, including 4,719 males and 4,613 females, diagnosed with Type 2 diabetes during a total of 293,238 person-years of follow-up. The mean follow-up for the development dataset was 2.7 years (SD 2.4, range 0-10 years). In the validation dataset, there were 3,783 patients, including 1,893 males and 1,890 females, diagnosed with Type 2 diabetes during a total of 115,113 person-years of follow-up. The mean follow-up for the validation dataset was 2.5 years (SD 2.3, range 0-10 years).

Baseline characteristics

Table 2 shows the baseline characteristics of patients in the development and validation datasets and for patients with no missing data. The distributions of continuous variables in the development and validation datasets are shown in Supplementary Figure S2.

For peer review only - http://bmjopen.bmj.com/site/about/guidelines.xhtml

Table 2. Characteristics of cohort at the index date in total, by number of missing variables, and by dataset.

			Missing variables		Dataset	
		Total	One or more	None	Development	Validation
Total		N=154,705	N=91,409	N=63,296	N=109,077	N=45,628
Age (years)		64.9 (14.2)	64.2 (14.9)	65.9 (13.1)	64.8 (14.2)	65.0 (14.2)
Sex	Male	71,063 (45.9%)	40,518 (44.3%)	30,545 (48.3%)	50,049 (45.9%)	21,014 (46.1%)
	Female	83,642 (54.1%)	50,891 (55.7%)	32,751 (51.7%)	59,028 (54.1%)	24,614 (53.9%)
Ethnicity	Non-white	14,116 (12.4%)	6,683 (13.3%)	7,433 (11.7%)	10,239 (12.9%)	3,877 (11.2%)
	White	99,468 (87.6%)	43,605 (86.7%)	55,863 (88.3%)	68,870 (87.1%)	30,598 (88.8%)
	Unknown	41,121	41,121	0	29,968	11,153
Current alcohol user		31,722 (20.5%)	14,867 (16.3%)	16,855 (26.6%)	22,320 (20.5%)	9,402 (20.6%)
Current smoker		21,126 (13.7%)	11,677 (12.8%)	9,449 (14.9%)	14,861 (13.6%)	6,265 (13.7%)
Medication	Antihypertensives	90,005 (58.2%)	47,424 (51.9%)	42,581 (67.3%)	63,290 (58.0%)	26,715 (58.5%)
	Atypical antipsychotics	3,959 (2.6%)	2,541 (2.8%)	1,418 (2.2%)	2,845 (2.6%)	1,114 (2.4%)
	Aspirin	41,986 (27.1%)	22,404 (24.5%)	19,582 (30.9%)	29,726 (27.3%)	12,260 (26.9%)
	Corticosteroids	55,090 (35.6%)	33,167 (36.3%)	21,923 (34.6%)	38,918 (35.7%)	16,172 (35.4%)
	Statins	74,166 (47.9%)	39,425 (43.1%)	34,741 (54.9%)	52,393 (48.0%)	21,773 (47.7%)
Medical/family history	Schizophrenia/bipolar	2,093 (1.4%)	1,189 (1.3%)	904 (1.4%)	1,493 (1.4%)	600 (1.3%)
	Cardiovascular disease	18,483 (11.9%)	9,608 (10.5%)	8,875 (14.0%)	12,862 (11.8%)	5,621 (12.3%)
	Depression	42,364 (27.4%)	26,066 (28.5%)	16,298 (25.7%)	29,627 (27.2%)	12,737 (27.9%)
	Learning disability	744 (0.5%)	446 (0.5%)	298 (0.5%)	478 (0.4%)	266 (0.6%)
	Diabetes in family	195 (0.1%)	117 (0.1%)	78 (0.1%)	159 (0.1%)	36 (0.1%)
	PCOS	840 (0.5%)	595 (0.7%)	245 (0.4%)	576 (0.5%)	264 (0.6%)
	Gestational diabetes	762 (0.5%)	592 (0.6%)	170 (0.3%)	567 (0.5%)	195 (0.4%)
	Renal/kidney disease	17,126 (11.1%)	9,109 (10.0%)	8,017 (12.7%)	11,810 (10.8%)	5,316 (11.7%)
	Sleep apnoea	2,289 (1.5%)	1,317 (1.4%)	972 (1.5%)	1,594 (1.5%)	695 (1.5%)
Clinical measures	HbA1c (mmol/mol)	43.5 (1.5)	43.5 (1.5)	43.5 (1.5)	43.5 (1.5)	43.5 (1.5)
	Cholesterol (mmol/L)	5.2 (1.2)	5.3 (1.2)	5.2 (1.2)	5.2 (1.2)	5.2 (1.2)
	Systolic BP (mmHg)	138.1 (18.5)	137.8 (18.8)	138.2 (18.4)	138.0 (18.6)	138.2 (18.5)
	Diastolic BP (mmHg)	80.0 (11.0)	79.6 (11.0)	80.2 (11.0)	79.9 (11.0)	80.1 (10.9)

BP=blood pressure. PCOS= Polycystic ovarian syndrome. Continuous variables are given as the mean (SD). Categorical variables are given as the number (%).Index of multiple deprivation, BMI, pulse, liver function test, and waist circumference are not included in the table since these measures are not available for >33.3% of the cohort.

For peer review only - http://bmjopen.bmj.com/site/about/guidelines.xhtml

The development dataset included 54.1% female and 12.9% non-white ethnicity; corresponding values in the validation dataset were 53.9% and 11.2%. Within the development dataset, 20.5% of patients were current alcohol users and 13.6% were current smokers compared with 20.6% and 13.7%, respectively, within the validation dataset. The percentage of patients with prescriptions of each medication was similar between the development and validation datasets. The most commonly prescribed medication was antihypertensives (58.0% in the development and 58.5% in the validation dataset), while the least common was atypical antipsychotics (2.6% and 2.4%, respectively). Of the 38,918 patients prescribed corticosteroids in the development dataset, 10,711 (27.5%) were prescribed oral medication, 19,192 were non-oral (49.3%), and 9,015 were prescribed both (23.2%; data not shown). For the validation dataset, there were 16,172 patients prescribed corticosteroids including 4,637 (28.7%) oral, 7,781 (48.1%) non-oral, and 3,754 prescribed both (23.2%). The medical/family history was similar between the development and validation datasets. The most common medical/family history condition was depression (27.2% in the development and 27.9% in the validation dataset), while the least common was a family history of diabetes (0.1% in both datasets). The mean HbA1c at the index date was the same for development and validation patients, 43.5mmol/mol (SD 1.2) or 6.1% (0.1%). Further, observed cholesterol and blood pressure were similar between the development and validation datasets.

Incidence rates of Type 2 diabetes

Supplementary Table S3 shows the incidence of Type 2 diabetes in total and in the development and validation datasets. The total incidence of Type 2 diabetes was 32.1 (95% CI 31.6-32.7) per 1,000 person-years (py): 31.8 (95% CI 31.2-32.5) in the development and 32.9 (95% CI 31.8-33.9) in the validation dataset. The largest rate difference between the development and validation datasets was for patients with a history of learning disability; the rate was 30.0 (95% CI 21.1-42.7) per 1,000 py in the development dataset compared with 41.2 (95% CI 27.6-61.5) in the validation dataset.

For peer review only - http://bmjopen.bmj.com/site/about/guidelines.xhtml

Predictor variables

Variables missing for more than 33.3% of the study cohort were eliminated as potential predictor variables including waist circumference (missing for 99.3% of patients), liver function test (99.2% missing), pulse rate (86.5% missing), BMI (73.6% missing), and deprivation (41.1% missing).

For flexible parametric modelling, three degrees of freedom were selected for the restricted cubic spline function used for the baseline hazard (AIC= 81,482, BIC= 81,520). This places two knots at percentile positions 33 and 67 of the distribution of the uncensored log survival times. Linear was the best fit for all continuous potential predictor variables; no fractional polynomial transformations were selected. Imputation did not significantly alter the distribution of cholesterol, blood pressure, and ethnicity (Supplementary Table S4).

The following potential predictor variables were removed during the backwards selection process: atypical antipsychotics, cholesterol, history of a learning disability, a history of depression, a history of schizophrenia or bipolar affective disorder, and ethnicity. The final male model comprised 14 predictor variables including HbA1c, systolic blood pressure, diastolic blood pressure, age, smoking, alcohol use; prescribed medications: antihypertensives, aspirin, corticosteroids, statins; and medical history of: cardiovascular disease, renal/kidney disease, sleep apnoea; and family history of diabetes (Table 3). The female model included two additional predictors, medical history of polycystic ovarian syndrome and gestational diabetes (Table 3).

For peer review only - http://bmjopen.bmj.com/site/about/guidelines.xhtml

Table 3. Development and final coefficients for the male and female prognostic models.

Predictor	Male					Female				
	Development model				Final model	Development model				Final model
	Coefficient	95% CI		p value	Coefficient	Coefficient	95% CI		p value	Coefficient
HbA1c (mmol/mol)	0.35048	0.33231	0.36866	0.000	0.34124	0.38494	0.36673	0.40315	0.000	0.38255
Age	-0.00310	-0.00579	-0.00040	0.024	-0.00302	-0.00465	-0.00737	-0.00193	0.001	-0.00462
Current alcohol user	0.05866	-0.00659	0.12391	0.078	0.05711	0.03588	-0.03874	0.11050	0.346	0.03566
Current smoker	-0.13053	-0.21393	-0.04714	0.002	-0.12709	-0.11355	-0.20407	-0.02302	0.014	-0.11284
Antihypertensive	0.13787	-0.03490	0.31064	0.118	0.13423	0.23830	-0.01509	0.49169	0.065	0.23682
Aspirin	0.10917	0.04131	0.17703	0.002	0.10629	0.13078	0.06142	0.20015	0.000	0.12997
Corticosteroids	0.13683	0.07441	0.19926	0.000	0.13322	0.12593	0.05951	0.19234	0.000	0.12515
Statins	0.65113	0.58046	0.72180	0.000	0.63396	0.66886	0.60170	0.73603	0.000	0.66471
Cardiovascular disease	-0.08578	-0.16955	-0.00201	0.045	-0.08352	-0.11919	-0.22249	-0.01590	0.024	-0.11845
Diabetes in family	0.65379	0.10842	1.19917	0.019	0.63655	0.37641	-0.31827	1.07110	0.288	0.37408
Polycystic ovarian syndrome	-	-	-	-	-	0.22766	-0.08223	0.53755	0.150	0.22625
Gestational diabetes	-	-	-	-	-	0.49865	0.24068	0.75661	0.000	0.49555
Renal/kidney disease	-0.05138	-0.15758	0.05481	0.343	-0.05003	-0.13741	-0.23253	-0.04229	0.005	-0.13655
Sleep apnoea	0.08901	-0.09730	0.27532	0.349	0.08666	0.35832	0.04615	0.67048	0.024	0.35609
Systolic blood pressure (mmHg)	0.00594	0.00383	0.00805	0.000	0.00578	0.00599	0.00347	0.00852	0.000	0.00596
Diastolic blood pressure (mmHg)	0.00359	0.00009	0.00708	0.044	0.00349	0.00053	-0.00333	0.00439	0.784	0.00053
Restricted cubic spline 1	0.96661	0.94161	0.99160	0.000	0.96661	0.93046	0.90612	0.95481	0.000	0.93046
Restricted cubic spline 2	-0.03565	-0.05114	-0.02016	0.000	-0.03565	-0.02957	-0.04468	-0.01445	0.000	-0.02957
Restricted cubic spline 3	0.03708	0.02516	0.04901	0.000	0.03708	0.01933	0.00740	0.03127	0.002	0.01933
Constant	-19.55409	-20.40687	-18.70131	0.000	-19.55409	-20.84774	-21.70300	-19.99247	0.000	-20.84774

Final model coefficients include adjustment for over-fitting.

For peer review only - http://bmjopen.bmj.com/site/about/guidelines.xhtml

Calibration

Using the developed model, Supplementary Figure S3 shows an example of the calibration between expected and observed probabilities of developing Type 2 diabetes at 10 years of follow up within one of the imputed female and male validation datasets. There were slight differences between plots from the different imputed datasets due to the different values imputed for predictors. Using Rubin's rules to combine the results across imputed datasets, the calibration slope was 0.974 (95% CI 0.905-1.042) for males and 0.994 (95% CI 0.931-1.057) for females. This indicates that the developed models were slightly overfitted. A uniform shrinkage factor (S=0.974 for males and S=0.994 for females) was applied to each developed model's beta coefficients before recalibrating the baseline function of the final model.

Discrimination

There was relatively good separation, or discrimination, between risk groups for both males and females when the developed models were fitted using the validation dataset. Supplementary Figure S4 shows an example using one of the imputed validation datasets. There were slight differences between plots from the different imputed datasets due to the different values imputed for predictors. For both males and females, the log-rank test for all imputed datasets indicated that the survivor functions were different between risk groups ($p<0.001$ for both males and females). Furthermore, validation showed that the male model discriminated reasonably well with mean Harrell's C statistic across imputed datasets of 0.701 and Somers' D statistic of 0.402; for the female model, the corresponding statistics were 0.718 and 0.436 (Table 4). These values suggest slightly better discrimination for the female model.

For peer review only - http://bmjopen.bmj.com/site/about/guidelines.xhtml

Table 4. Male and female prognostic model mean performance statistics across imputed datasets.

Measure	Male		Female	
	Development	Validation	Development	Validation
Harrell's C	0.700	0.701	0.720	0.718
Somers' D	0.401	0.402	0.441	0.436
Calibration slope	1.000	0.974	1.000	0.994

For peer review only

For peer review only - http://bmjopen.bmj.com/site/about/guidelines.xhtml

DISCUSSION

Although several prognostic and diagnostic models for predicting Type 2 diabetes-related outcomes have been developed and validated within the UK, none to date has been specifically developed in a population with NDH, for whom the risk profile is likely different than the general population. The available evidence shows that the incidence of Type 2 diabetes in the cohort of patients used to develop the QDiabetes-2018 risk assessment tool was 4.17 (95% CI 4.15 to 4.19) per 1,000 person-years (17). Those included in our study were significantly more likely to develop Type 2 diabetes. In fact, the incidence in our development cohort was nearly eight times that of the QDiabetes-2018 development cohort. Therefore, we have developed and validated pragmatic sex-specific prognostic models for predicting the risk of developing Type 2 diabetes in those with NDH, which could be used for targeting referral to the NHS DPP. Our models include important risk factors for people that already have NDH.

Since the primary aim of this study was to develop models that could be easily implemented using routinely collected data, in the variable selection process we closely considered data availability and excluded variables with high levels of missing data, including waist circumference, liver function, pulse rate, BMI, and deprivation. Waist circumference and BMI are key risk factors for Type 2 diabetes, but these measures may not be obtained due to lack of time and other practical or perceived barriers (25). BMI, in particular, has been included in many existing Type 2 diabetes models (10). However, the inclusion of BMI must be balanced with practicality, given that our data showed BMI (or height and weight) were infrequently recorded in a primary care setting.

Since the models were developed using observational primary care data, the accuracy of coding, particularly of the outcome, has the potential to affect model development. Research published in 2011 found that miscoding, misdiagnosis, and misclassification of diabetes was common in UK primary care (26). However, in more recent years, implementation of the UK Quality and Outcomes Framework

For peer review only - http://bmjopen.bmj.com/site/about/guidelines.xhtml

(QoF) has resulted in better coding of Type 2 diabetes, specifically within CPRD (27, 28). With improved interoperability, the launch of SNOMED is expected to further boost coding accuracy (29). Since this research utilised data initially recorded for managing the care of individual patients, there are also a number of potential sources of bias. To address this, the study cohort included only patients that are considered by CPRD of acceptable research standards. Further, clinical measures that were not biologically plausible and likely misreported were excluded. In most cases, another value that was biologically plausible was available within the same period for the patient.

This study has several strengths. These models are for use in primary care. Therefore, we used a primary care database (CPRD) to develop the models. In recent years the HbA1c assay has been the preferred method to diagnose NDH and Type 2 diabetes compared with oral glucose tolerance or fasting plasma glucose tests (30). Therefore, these models were developed using HbA1c to quantify blood glucose. The large sample size allowed for a sufficient number of events per predictor parameter. We considered a range of predictors specifically selected due to clinical relevance to development of Type 2 diabetes. Continuous predictors were not categorised, so there was no loss of information. The decision to develop sex-specific models was based on the presence of some sex-specific risk factors, like history of gestational diabetes. Additionally, we identified new risk factors not included in the 2018 update of QDiabetes, which was developed within the general population (17). These risk factors include history of sleep apnoea, blood pressure, alcohol use, prescription of antihypertensives, and prescription of aspirin.

This study also had several limitations. The primary limitation is the splitting of the cohort into development and validation datasets instead of using a fully external database to validate the model. However, given the size of the cohort and the large number of events, this likely had little effect on model development. Furthermore, to ensure case mix, non-random selection was used to split the cohort. The outcome for this study was defined using a single medcode or test result indicating Type

For peer review only

2 diabetes. In practice, this would typically be confirmed via a follow up test. Another limitation is that the models included predictor variables obtained at one point in time including a single HbA1c measure to determine NDH. However, the models could be adjusted to include time-varying predictors relatively easily. Methods such as land marking or joint models could be used to model changes in predictors over time. Some predictor variables were self-reported including smoking, alcohol use, and family history of diabetes. The proportion of non-current smokers is in line with a similar study while the proportion of patients with a family history of diabetes in this study was much lower than that reported in a similar study.(17) This may indicate that family history of diabetes is not established in clinical practice or established but not recorded within the CPRD. Prescriptions issued were used as a proxy for current medication. Patients may not have filled the prescription or adhered to the medication. Because this was an open cohort and the number of people diagnosed with NDH has increased in recent years, the mean follow-up time was short- 2.7 years for patients in the development dataset and 2.5 years for patients in the validation dataset. However, 14,896 patients in the development dataset and 5,678 patients in the validation dataset had five or more years of follow up. Therefore, based on existing research, we believe that there was sufficient follow-up time to determine risk for progression to Type 2 diabetes. HES and ONS linkage was only available for 59.0% of patients in the cohort. If linkage to ONS was not available and a date of death was provided in CPRD, then the CPRD date was used. While ONS is the gold standard for date of death, deaths are less well coded in CPRD. It is possible that deaths for some patients without linkage to ONS were never coded in CPRD, and the patients were not censored accordingly. However, this likely only affected a few patients. It is possible that patients receiving non-metformin oral hypoglycaemic agents at baseline were included in the cohort. However, it is highly unlikely that a patient would have been prescribed a non-metformin oral hypoglycaemic agent without meeting any of the other exclusion criteria. Finally, there may have been additional predictor variables that were not considered either because they are not collected as part of routine clinical care or because they are not among the known traditional risk factors for Type 2 diabetes.

For peer review only - http://bmjopen.bmj.com/site/about/guidelines.xhtml

For peer review only

Similar to the QRISK cardiovascular disease risk algorithm, the models presented are designed to be integrated into primary care computer systems to automatically calculate risk (31). At the time of the first HbA1c test indicating NDH, a risk score could be automatically generated using the HbA1c measure along with clinical, prescription, and diagnoses data already contained in the individual's electronic health record. Additionally, the algorithm for imputing missing data could also be implemented automatically. Rather than referring all adults with NDH to the NHS DPP, healthcare providers could prioritize referrals for people at high risk for progressing to Type 2 diabetes.

The NHS DPP is a limited resource and does not have current capacity to accommodate all adults with NDH in England. People are referred to the NHS DPP through the NHS Health Check programme, aimed at people aged 40-74, or people with NDH identified through opportunistic assessment or as part of routine clinical care (9). Eligibility for the NHS DPP is typically determined through an HbA1c measure or, less frequently, an Oral Glucose Tolerance Test (OGTT). However, this study has identified additional factors to stratify further the risk of developing Type 2 diabetes within this high-risk group. Targeting referrals may be a more cost-effective and efficient way to deliver the NHS DPP. The male and female prognostic models we developed and validated could be used to identify and target those most at risk of developing Type 2 diabetes for referral to the NHS DPP. Implementation of these models would standardise the NHS DPP identification and referral process to be consistent across sites and based on information already collected as part of primary care. The next step is to determine the optimum risk threshold to accurately identify patients that will develop Type 2 diabetes.

For peer review only - http://bmjopen.bmj.com/site/about/guidelines.xhtml

Footnotes

CRediT Author Statement

Conceptualization: LJG, FZ, MJD, KK; funding acquisition: LJG (lead), MJD, FZ; methodology, writing – original draft: BC, LJG; data curation, formal analysis, validation, visualization: BC; software: BC, SB; writing – review & editing: FZ, MJD, KK, SB. All authors provided final approval of the version to publish. The corresponding author (BC) had full access to all the data in the study and had final responsibility for the decision to submit it for publication.

Competing interests

BC, LJG, FZ, and SB: none
MJD has acted as consultant, advisory board member and speaker for Novo Nordisk, Sanofi-Aventis, Lilly, Merck Sharp & Dohme, Boehringer Ingelheim, AstraZeneca and Janssen, an advisory board member for Servier and as a speaker for Mitsubishi Tanabe Pharma Corporation and Takeda Pharmaceuticals International Inc. She has received grants in support of investigator and investigator initiated trials from Novo Nordisk, Sanofi-Aventis, Lilly, Boehringer Ingelheim and Janssen. She was a member of the NICE public health guideline for prevention of Type 2 diabetes (NICE PH 38).
KK has acted as a consultant and speaker for Novartis, Novo Nordisk, Sanofi-Aventis, Lilly and Merck Sharp & Dohme. He has received grants in support of investigator and investigator-initiated trials from Novartis, Novo Nordisk, Sanofi-Aventis, Lilly, Pfizer, Boehringer Ingelheim and Merck Sharp & Dohme. He is a member of the External Reference Group of the NHS DPP and was Chair of the NICE public health guideline for prevention of Type 2 diabetes (NICE PH 38).

Acknowledgements

This study is based on data from the Clinical Practice Research Datalink GOLD database obtained under licence from the UK Medicines and Healthcare products Regulatory Agency. However, the interpretation and conclusions contained in this article are those of the authors alone.

The authors gratefully acknowledge Leicester Real-World Evidence Unit (LRWE) for facilitating the download of CPRD data. LRWE is funded by University of Leicester, National Institute for Health Research (NIHR) Applied Research Collaboration East Midlands (ARC EM) and NIHR Leicester Biomedical Research Centre. The interpretation and conclusions contained in this report/article do not necessarily reflect those of the LRWE.

Funding

This research was supported by a grant from National Institute for Health Research (NIHR) Collaboration for Leadership in Applied Health Research and Care (CLAHRC) East Midlands. The funding body had no role in the study design, data collection, analysis, or interpretation; in the writing of the manuscript; or in the decision to submit the manuscript.

For peer review only

For peer review only - http://bmjopen.bmj.com/site/about/guidelines.xhtml

This research was also funded in part by the NIHR Leicester Biomedical Research Centre and NIHR Applied Research Collaboration. The views expressed are those of the author(s) and not necessarily those of the NHS, the NIHR or the Department of Health and Social Care.

Ethical approval

This research was approved by the Independent Scientific Advisory Committee (ISAC) for Medicines and Healthcare products Regulatory Agency Database Research (protocol 18_238).

Data sharing

Patient-level electronic health records obtained from CPRD cannot be shared. However, the authors will share programming code and aggregate statistics if requested. A list of medcodes used to define Type 2 diabetes, pre-existing Type 1 diabetes, and medical and family history as well as product codes used to identify current medication is available at https://github.com/bc188/Prognostic-model-codes.

For peer review only

For peer review only - http://bmjopen.bmj.com/site/about/guidelines.xhtml

REFERENCES

1. Barron E, Valabhji J, Young B. Prevalence, characteristics, distribution and identification of non-diabetic hyperglycaemia in England, 2016. Available from https://wwwgovuk/government/news/five-million-people-at-high-risk-of-type-2-diabetes. Accessed 20 Sept 2019
2. Gillies CL, Abrams KR, Lambert PC, Cooper NJ, Sutton AJ, Hsu RT, Khunti K. Pharmacological and lifestyle interventions to prevent or delay type 2 diabetes in people with impaired glucose tolerance: systematic review and meta-analysis. BMJ 2007;334:299
3. Kramer MK, Miller RG, Siminerio LM Evaluation of a community Diabetes Prevention Program delivered by diabetes educators in the United States: One-year follow up. Diabetes Res Clin Pr 2014;106(3):E49-E52.
4. Knowler WC B-CE, Fowler SE, et al. Reduction in the incidence of type 2 diabetes with lifestyle intervention or metformin. N Engl J Med 2002; 346:393-403
5. Tuomilehto J, Lindström J, Eriksson JG, Valle TT, Hämäläinen H, Ilanne-Parikka P, Keinänen-Kiukaanniemi S, Laakso M, Louheranta A, Rastas M, Salminen V, Uusitupa M. Prevention of Type 2 Diabetes Mellitus by Changes in Lifestyle among Subjects with Impaired Glucose Tolerance. N Engl J Med 2001; 344:1343-1350
6. Pan XR, Li GW, Hu YH, Wang JX, Yang WY, An ZX, Hu ZX, Lin J, Xiao JZ, Cao HB, Liu PA, Jiang XG, Jiang YY, Wang JP, Zheng H, Zhang H, Bennett PH, Howard BV. Effects of diet and exercise in preventing NIDDM in people with impaired glucose tolerance: the Da Qing IGT and Diabetes Study. Diabetes Care 1997; 20:537-544
7. Troughton J, Chatterjee S, Hill SE, Daly H, Martin Stacey L, Stone MA, Patel N, Khunti K, Yates T, Gray LJ, Davies MJ. Development of a lifestyle intervention using the MRC framework for diabetes prevention in people with impaired glucose regulation. J Public Health (Oxf) 38: 493-501
8. England NHS. Roll out of the Programme, 2016. Available from https://www.england.nhs.uk/diabetes/diabetes-prevention/roll-out-of-the-programme. Accessed 20 Sept 2019
9. England NHS. NHSDPP overview and FAQ, 2016. Available from https://www.england.nhs.uk/wp-content/uploads/2016/08/dpp-faq.pdf. Accessed 20 Sept 2019
10. Collins GS, Mallett S, Omar O, Yu L. Developing risk prediction models for type 2 diabetes: a systematic review of methodology and reporting. BMC Med 2011;9:103
11. Noble D, Mathur R, Dent T, Meads C, Greenhalgh T. Risk models and scores for type 2 diabetes: systematic review. BMJ 2011;343(d7163).
12. Barber SR, Davies MJ, Khunti K, Gray LJ. Risk assessment tools for detecting those with pre-diabetes: a systematic review. Diabetes Res Clin Pract 2014;105:1-13
13. Bodicoat DH, Khunti K, Srinivasan BT, Mostafa S, Gray LJ, Davies MJ, Webb DR. Incident Type 2 diabetes and the effect of early regression to normoglycaemia in a population with impaired glucose regulation. Diabet Med 2017;34:396-404
14. Twohig H, Hodges V, Mitchell C. Pre-diabetes: opportunity or overdiagnosis?. *Br J Gen Pract*. 2018;68(669):172-173.
15. Herrett E, Gallagher AM, Bhaskaran K, Forbes H, Mathur R, van Staa T, Smeeth L. Data resource profile: clinical practice research datalink (CPRD). Int J Epidemiol 2015;44:827-36
16. Riley RD, Snell KI, Ensor J, Burke DL, Harrell Jr FE, Moons KG, Collins GS. Minimum sample size for developing a multivariable prediction model: PART II-binary and time-to-event outcomes. Stat Med 2019;38:1276-96
17. Hippisley-Cox J, Coupland C. Development and validation of QDiabetes-2018 risk prediction algorithm to estimate future risk of type 2 diabetes: cohort study. BMJ 2017;359:j5019
18. Gray LJ, Taub N, Khunti K, Gardiner E, Hiles S, Webb DR, Srinivasan BT, Davies MJ. The Leicester Risk Assessment score for detecting undiagnosed Type 2 diabetes and impaired glucose regulation for use in a multiethnic UK setting. Diabet Med 2010;27:887-95
19. Van der Heijden GJ, Donders ART, Stijnen T, Moons KG. Imputation of missing values is superior to complete case analysis and the missing-indicator method in multivariable diagnostic research: a clinical example. J Clin Epidemiol 2006;59:1102-9
20. Royston P, Lambert PC. Flexible parametric survival analysis using Stata: beyond the Cox model. Stat Med 2014;33:5280-97
21. Dunkler D, Plischke M, Leffondré K, Heinze G. Augmented backward elimination: a pragmatic and purposeful way to develop statistical models. PLoS One 2014;9:e113677

For peer review only - http://bmjopen.bmj.com/site/about/guidelines.xhtml

22. Harrell FE, Califf RM, Pryor DB, Lee KL, Rosati RA. Evaluating the yield of medical tests. JAMA 1982;247:2543-6
23. Harrell FE, Lee KL, Mark DB. Multivariable prognostic models: Issues in developing models, evaluating assumptions and adequacy, and measuring and reducing errors. Stat Med 1996;15:361-87
24. Snee R. Validation of Regression Models: Methods and Examples. Technometrics 1977;19:419-28
25. Dunkley AJ, Stone MA, Patel N, Davies MJ, Khunti K. Waist circumference measurement: knowledge, attitudes and barriers in patients and practitioners in a multi-ethnic population. Fam Pract 2009;26:365-71
26. De Lusignan S, Sadek N, Mulnier H, Tahir A, Russell-Jones D, Khunti K. Miscoding, misclassification and misdiagnosis of diabetes in primary care. Diabet Med 2012;29:181-9
27. Calvert M, Shankar A, McManus RJ, Lester H, Freemantle N. Effect of the quality and outcomes framework on diabetes care in the United Kingdom: retrospective cohort study. BMJ 2009;338:b1870
28. Tate AR, Dungey S, Glew S, Beloff N, Williams R, Williams T. Quality of recording of diabetes in the UK: how does the GP's method of coding clinical data affect incidence estimates? Cross-sectional study using the CPRD database. BMJ open 2017;7:e012905
29. Haarbrandt B, Schreiweis B, Rey S, Sax U, Scheithauer S, Rienhoff O, Knaup-Gregori P, Bavendiek U, Dieterich C, Brors B, Kraus I, Thoms CM, Jäger D, Ellenrieder V, Bergh B, Yahyapour R, Eils R, Consortium H, Marschollek M. HiGHmed - An Open Platform Approach to Enhance Care and Research across Institutional Boundaries. Methods Inf Med 2018;57:e66-e81
30. International Expert Committee. International Expert Committee report on the role of the A1C assay in the diagnosis of diabetes. Diabetes Care 2009;32:1327-34
31. Hippisley-Cox J, Coupland C, Brindle P. Development and validation of QRISK3 risk prediction algorithms to estimate future risk of cardiovascular disease: prospective cohort study. BMJ. 2017;357:j2099

For peer review only

For peer review only - http://bmjopen.bmj.com/site/about/guidelines.xhtml

Supplementary Table S1. Number of practices by region in total and included in the development and validation datasets.

Practice region	Total	Dataset	
		Development	Validation
North East	11	8	3
North West	85	60	26
Yorkshire & The Humber	28	20	8
East Midlands	25	18	8
West Midlands	61	43	18
East of England	54	38	16
South West	61	43	18
South Central	56	39	17
London	95	67	29
South East Coast	68	48	20
Northern Ireland	25	18	8
Scotland	94	66	28
Wales	77	54	23
**Total**	**740**	**518**	**222**

For peer review only

Supplementary Table S2. Percent of patients missing potential predictor variables.

Predictor variable	Missing n	Missing %
Waist circumference	153,592	99.3
Liver function test	153,493	99.2
Pulse rate	133,890	86.5
BMI	113,840	73.6
Index of Multiple Deprivation	63,524	41.1
Systolic blood pressure	48,390	31.3
Diastolic blood pressure	48,390	31.3
Ethnicity	41,121	26.6
Serum cholesterol	38,910	25.2
HbA1c	0	0
Age	0	0
Sex	0	0
Current alcohol use	0	0
Current smoker	0	0
Antihypertensives	0	0
Atypical antipsychotics	0	0
Aspirin	0	0
Corticosteroids	0	0
Statins	0	0
Bipolar disease or schizophrenia	0	0
Cardiovascular disease	0	0
Depression	0	0
Learning disability	0	0
Diabetes in family	0	0
Polycystic ovarian syndrome	0	0
Gestational diabetes	0	0
Renal/kidney disease	0	0
Sleep apnoea	0	0

For peer review only

Supplementary Table S3. Incidence of Type 2 diabetes per 1,000 person years with 95% confidence intervals.

					Dataset					
		Total			Development			Validation		
		Py	n	Rate (95% CI)	Py	n	Rate (95% CI)	Py	n	Rate (95% CI)
Total		408,350.5	13,115	32.1 (31.6-32.7)	293,237.8	9,332	31.8 (31.2-32.5)	115,112.6	3,783	32.9 (31.8-33.9)
Age group	<30	4,285.1	79	18.4 (14.8-23.0)	3,017.0	56	18.6 (14.3-24.1)	1,268.1	23	18.1 (12.1-27.3)
	30-39	15,214.7	307	20.2 (18.0-22.6)	11,050.8	231	20.9 (18.4-23.8)	4,164.0	76	18.3 (14.6-22.9)
	40-49	43,354.3	1,157	26.7 (25.2-28.3)	31,539.3	836	26.5 (24.8-28.4)	11,815.0	321	27.2 (24.4-30.3)
	50-59	81,437.4	2,399	29.5 (28.3-30.7)	58,691.3	1,730	29.5 (28.1-30.9)	22,746.1	669	29.4 (27.3-31.7)
	60-69	109,599.6	3,808	34.7 (33.7-35.9)	79,177.3	2,709	34.2 (32.9-35.5)	30,422.4	1,099	36.1 (34.1-38.3)
	70-79	96,100.4	3,553	37.0 (35.8-38.2)	68,493.3	2,527	36.9 (35.5-38.4)	27,607.1	1,026	37.2 (35.0-39.5)
	80-89	50,818.9	1,629	32.1 (30.5-33.7)	36,072.2	1,114	30.9 (29.1-32.8)	14,746.7	515	34.9 (32.0-38.1)
	90+	7,540.0	183	24.3 (21.0-28.1)	5,196.7	129	24.8 (20.9-29.5)	2,343.2	54	23.0 (17.6-30.1)
Sex	Male	186,953.5	6,612	35.4 (34.5-36.2)	134,390.2	4,719	35.1 (34.1-36.1)	52,563.3	1,893	36.0 (34.4-37.7)
	Female	221,397.0	6,503	29.4 (28.7-30.1)	158,847.6	4,613	29.0 (28.2-29.9)	62,549.3	1,890	30.2 (28.9-31.6)
Ethnicity	Non-white	38,606.0	1,154	29.9 (28.2-31.7)	29,281.3	863	29.5 (27.6-31.5)	9,324.7	291	31.2 (27.8-35.0)
	White	257,231.3	8,446	32.8 (32.1-33.5)	181,622.3	5,878	32.4 (31.5-33.2)	75,609.0	2,568	34.0 (32.7-35.3)
Current alcohol user	No	321,672.8	10,049	31.2 (30.6-31.9)	231,489.6	7,223	31.2 (30.5-31.9)	90,183.2	2,826	31.3 (30.2-32.5)
	Yes	86,677.6	3,066	35.4 (34.1-36.6)	61,748.2	2,109	34.2 (32.7-35.6)	24,929.4	957	38.4 (36.0-40.9)
Current smoker	No	351,866.5	11,355	32.3 (31.7-32.9)	252,907.8	8,103	32.0 (31.3-32.7)	98,958.7	3,252	32.9 (31.8-34.0)
	Yes	56,483.9	1,760	31.2 (29.7-32.6)	40,330.0	1,229	30.5 (28.8-32.2)	16,154.0	531	32.9 (30.2-35.8)
Antihypertensives	No	402,244.5	12,840	31.9 (31.4-32.5)	288,800.1	9,137	31.6 (31.0-32.3)	113,444.4	3,703	32.6 (31.6-33.7)
	Yes	6,105.9	275	45.0 (40.0-50.7)	4,437.7	195	43.9 (38.2-50.6)	1,668.3	80	48.0 (38.5-59.7)
Atypical antipsychotics	No	397,003.1	12,760	32.1 (31.6-32.7)	284,987.3	9,084	31.9 (31.2-32.5)	112,015.8	3,676	32.8 (31.8-33.9)
	Yes	11,347.4	355	31.3 (28.2-34.7)	8,250.5	248	30.1 (26.5-34.0)	3,096.9	107	34.6 (28.6-41.8)
Aspirin	No	282,265.5	7,971	28.2 (27.6-28.9)	202,397.8	5,686	28.1 (27.4-28.8)	79,867.7	2,285	28.6 (27.5-29.8)
	Yes	126,085.0	5,144	40.8 (39.7-41.9)	90,840.0	3,646	40.1 (38.9-41.5)	35,245.0	1,498	42.5 (40.4-44.7)
Corticosteroids	No	132,237.8	3,781	28.6 (27.7-29.5)	94,557.1	2,721	28.8 (27.7-29.9)	37,680.7	1,060	28.1 (26.5-29.9)
	Yes	276,112.7	9,334	33.8 (33.1-34.5)	198,680.8	6,611	33.3 (32.5-34.1)	77,431.9	2,723	35.2 (33.9-36.5)
Statins	No	197,618.7	4,184	21.2 (20.5-21.8)	141,932.3	2,977	21.0 (20.2-21.7)	55,686.3	1,207	21.7 (20.5-22.9)
	Yes	210,731.8	8,931	42.4 (41.5-43.3)	151,305.5	6,355	42.0 (41.0-43.0)	59,426.3	2,576	43.3 (41.7-45.1)
Schizophrenia/bipolar	No	402,889.4	12,937	32.1 (31.6-32.7)	289,246.4	9,212	31.8 (31.2-32.5)	113,642.9	3,725	32.8 (31.7-33.8)
	Yes	5,461.1	178	32.6 (28.1-37.8)	3,991.4	120	30.1 (25.1-36.0)	1,469.7	58	39.5 (30.5-51.0)
Cardiovascular disease	No	361,574.5	11,297	31.2 (30.7-31.8)	260,237.0	8,074	31.0 (30.4-31.7)	101,337.5	3,223	31.8 (30.7-32.9)
	Yes	46,776.0	1,818	38.9 (37.1-40.7)	33,000.8	1,258	38.1 (36.1-40.3)	13,775.1	560	40.7 (37.4-44.2)
Depression	No	303,786.2	9,875	32.5 (31.9-33.2)	219,040.2	7,043	32.2 (31.4-32.9)	84,746.0	2,832	33.4 (32.2-34.7)

For peer review only

	Yes	104,564.3	3,240	31.0 (29.9-32.1)	74,197.7	2,289	30.9 (29.6-32.1)	30,366.6	951	31.3 (29.4-33.4)
Learning disability	No	406,734.9	13,060	32.1 (31.6-32.7)	292,204.8	9,301	31.8 (31.2-32.5)	114,530.1	3,759	32.8 (31.8-33.9)
	Yes	1,615.6	55	34.0 (26.1-44.3)	1,033.0	31	30.0 (21.1-42.7)	582.5	24	41.2 (27.6-61.5)
Diabetes in family	No	407,867.0	13,091	32.1 (31.6-32.7)	292,821.8	9,311	31.8 (31.2-32.4)	115,045.1	3,780	32.9 (31.8-33.9)
	Yes	483.5	24	49.6 (33.3-74.1)	416.0	21	50.5 (32.9-77.4)	67.5	<5	44.4 (14.3-137.8)
Renal/kidney disease	No	368,309.2	11,766	31.9 (31.4-32.5)	265,292.2	8,403	31.7 (31.0-32.4)	103,016.9	3,363	32.6 (31.6-33.8)
	Yes	40,041.3	1,349	33.7 (31.9-35.5)	27,945.6	929	33.2 (31.2-35.5)	12,095.7	420	34.7 (31.6-38.2)
Sleep apnoea	No	403,300.5	12,896	32.0 (31.4-32.5)	289,628.0	9,178	31.7 (31.0-32.3)	113,672.4	3,718	32.7 (31.7-33.8)
	Yes	5,050.0	219	43.4 (38.0-49.5)	3,609.8	154	42.7 (36.4-50.0)	1,440.2	65	45.1 (35.4-57.6)
PCOS*	No	219,461.5	6,441	29.3 (28.6-30.1)	157,488.6	4,571	29.0 (28.2-29.9)	61,972.9	1,870	30.2 (28.8-31.6)
	Yes	1,935.5	62	32.0 (25.0-41.1)	1,359.0	42	30.9 (22.8-41.8)	576.5	20	34.7 (22.4-53.8)
Gestational diabetes*	No	219,205.1	6,423	29.3 (28.6-30.0)	157,163.0	4,550	29.0 (28.1-29.8)	62,042.1	1,873	30.2 (28.9-31.6)
	Yes	2,191.9	80	36.5 (29.3-45.4)	1,684.7	63	37.4 (29.2-47.9)	507.2	17	33.5 (20.8-53.9)
HbA1c (mmol/mol)	42	143,564.4	2,341	16.3 (15.7-17.0)	102,303.4	1,650	16.1 (15.4-16.9)	41,261.0	691	16.7 (15.5-18.0)
	43	103,706.7	2,496	24.1 (23.1-25.0)	74,289.4	1,762	23.7 (22.6-24.9)	29,417.3	734	25.0 (23.2-26.8)
	44	72,839.5	2,563	35.2 (33.9-36.6)	52,495.5	1,801	34.3 (32.8-35.9)	20,344.0	762	37.5 (34.9-40.2)
	45	48,523.8	2,407	49.6 (47.7-51.6)	35,497.9	1,724	48.6 (46.3-50.9)	13,025.9	683	52.4 (48.6-56.5)
	46	31,687.9	2,473	78.0 (75.0-81.2)	22,985.0	1,794	78.1 (74.5-81.7)	8,702.9	679	78.0 (72.4-84.1)
	47	8,028.2	835	104.0 (97.2-111.3)	5,666.6	601	106.1 (97.9-114.9)	2,361.6	234	99.1 (87.2-112.6)
Cholesterol (mmol/L)	<5.0	130,946.3	4,568	34.9 (33.9-35.9)	93,611.8	3,273	35.0 (33.8-36.2)	37,334.5	1,295	34.7 (32.8-36.6)
	5.0-6.9	152,342.9	4,978	32.7 (31.8-33.6)	109,313.4	3,548	32.5 (31.4-33.5)	43,029.5	1,430	33.2 (31.6-35.0)
	≥7.0	24,848.2	817	32.9 (30.7-35.2)	17,982.7	573	31.9 (29.4-34.6)	6,865.5	244	35.5 (31.3-40.3)
Systolic BP (mmHg)	<140	147,766.9	4,476	30.3 (29.4-31.2)	105,813.8	3,195	30.2 (29.2-31.3)	41,953.1	1,281	30.5 (28.9-32.3)
	≥140	135,710.1	5,206	38.4 (37.3-39.4)	97,560.5	3,685	37.8 (36.6-39.0)	38,149.6	1,521	39.9 (37.9-41.9)
Diastolic BP (mmHg)	<90	228,884.2	7,540	32.9 (32.2-33.7)	164,236.7	5,367	32.7 (31.8-33.6)	64,647.6	2,173	33.6 (32.2-35.1)
	≥90	54,592.8	2,142	39.2 (37.6-40.9)	39,137.6	1,513	38.7 (36.8-40.7)	15,455.2	629	40.7 (37.6-44.0)

BP=blood pressure. PCOS=Polycystic ovarian syndrome.
Table includes observed values only. Imputed values for ethnicity, serum cholesterol, and systolic and diastolic blood pressure are not included.
Index of multiple deprivation, BMI, pulse, liver function test, and waist circumference are not included in the table since these measures are not available for >33.3% of the cohort.
Age was collapsed into 10-year groups. HbA1c was collapsed into one mmol/mol increments. Cholesterol was collapsed into three clinically relevant groups (<5.0, 5.0-6.9, and ≥7.0mmol/L). Systolic blood pressure was collapsed into two clinically relevant groups based on NICE guidelines for hypertension (<140 and ≥140 mmHg) as was diastolic blood pressure (<90 and ≥90 mmHg) (27).
***The incidence was calculated among females only.**
[+]Note, n<5 cannot be published.

For peer review only

Supplementary Table S4. Descriptive statistics for cholesterol, blood pressure, and ethnicity for observed (non-missing) development data and final development data (including observed and imputed).

Variable	Dataset	Mean	Std. Dev.
Cholesterol (mmol/L)	Observed	5.23	1.19
	Observed+Imputed	5.26	1.19
Systolic blood pressure (mmHg)	Observed	138.02	18.57
	Observed+Imputed	137.80	18.59
Diastolic blood pressure (mmHg)	Observed	79.92	11.01
	Observed+Imputed	80.21	11.01
White Ethnicity (proportion)	Observed	0.87	0.34
	Observed+Imputed	0.87	0.33

Observed+Imputed comprises the final data. The distribution of the observed data with the observed+imputed data overlaid was visually examined and no large differences were seen.

For peer review only

Supplementary Figure S1. Participant flow diagram.

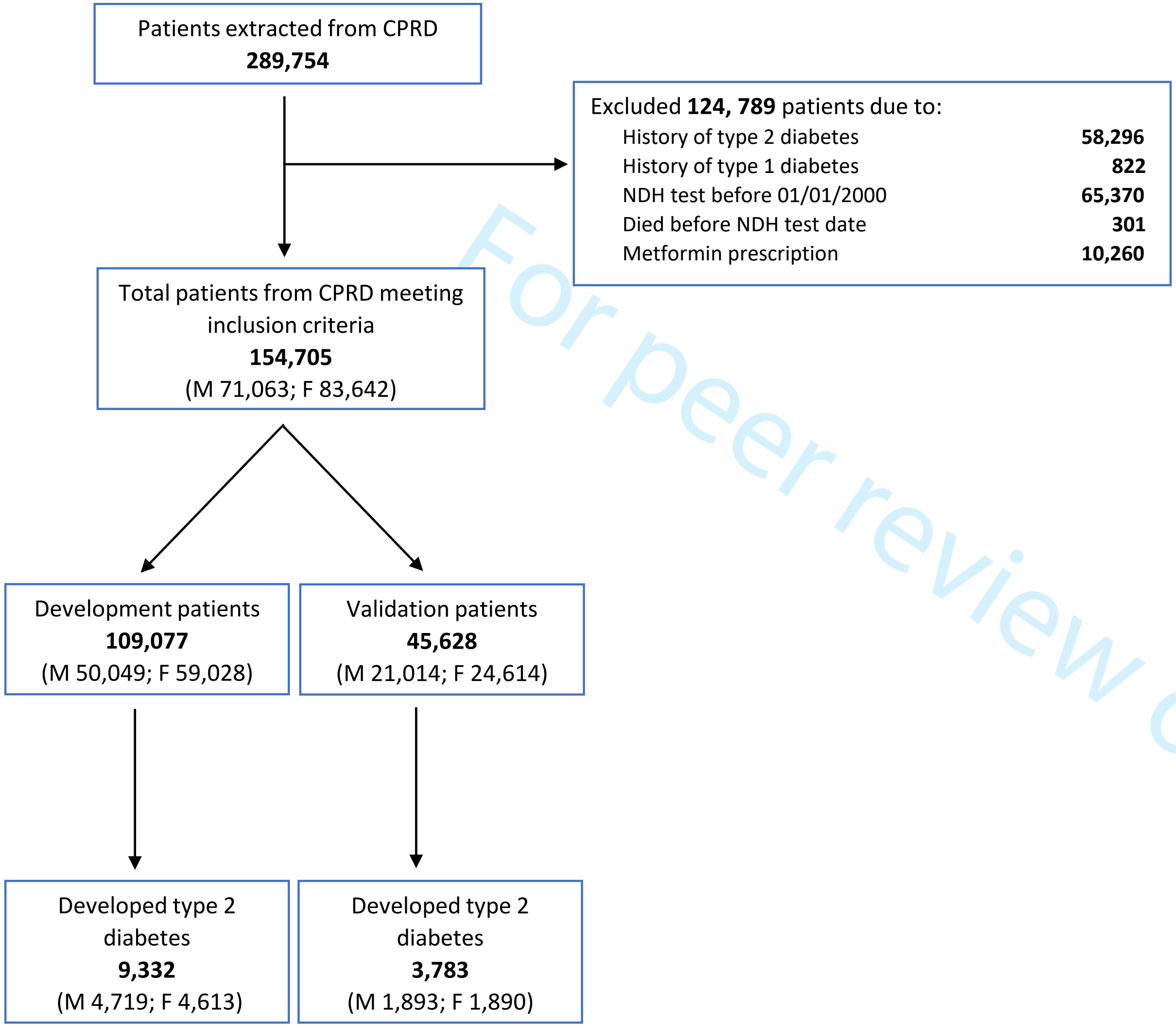

For peer review only

Supplementary Figure S2. Distribution of continuous variables for the development and validation datasets.

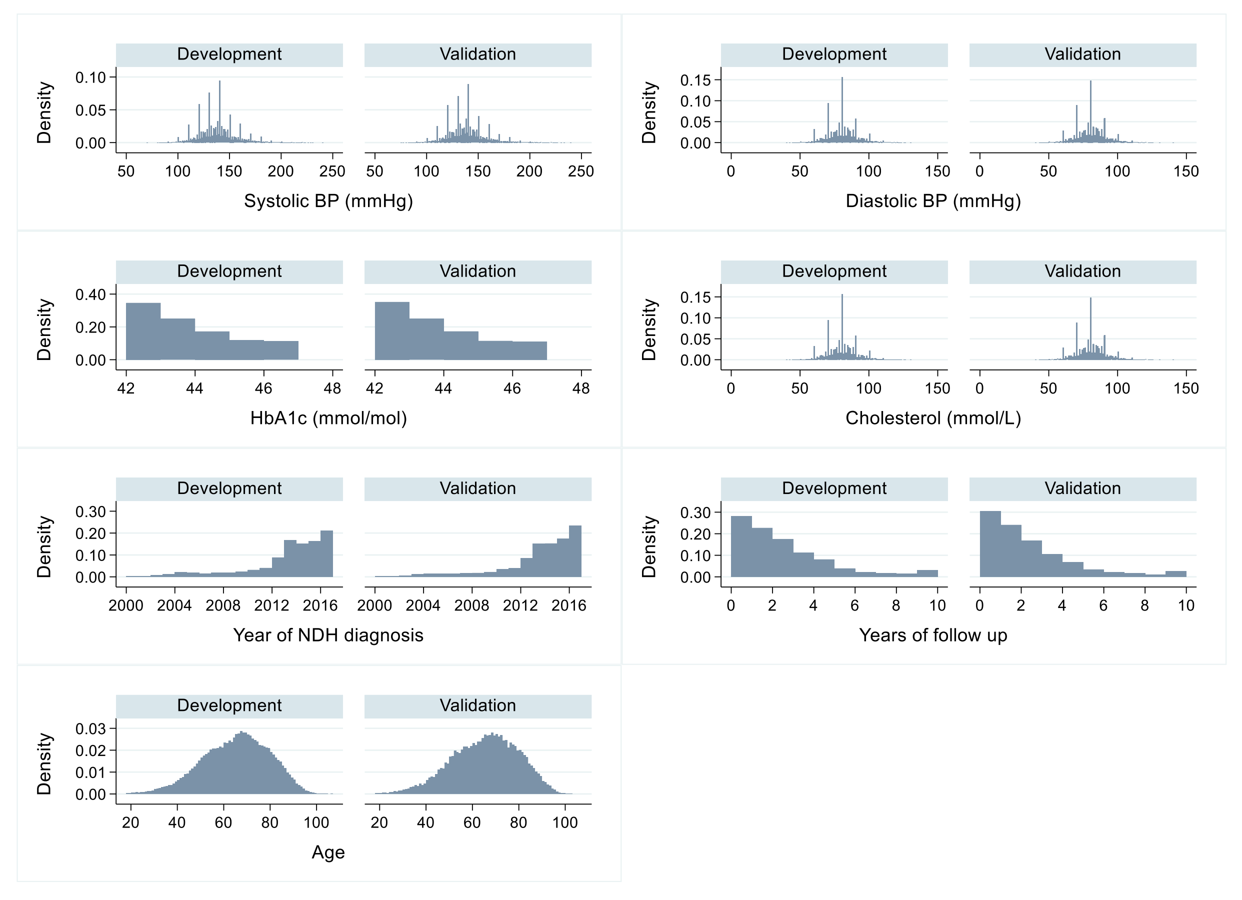

Supplementary Figure S3. Calibration plots by 10-year Type 2 diabetes risk deciles for the male and female models in one of the imputed validation datasets.

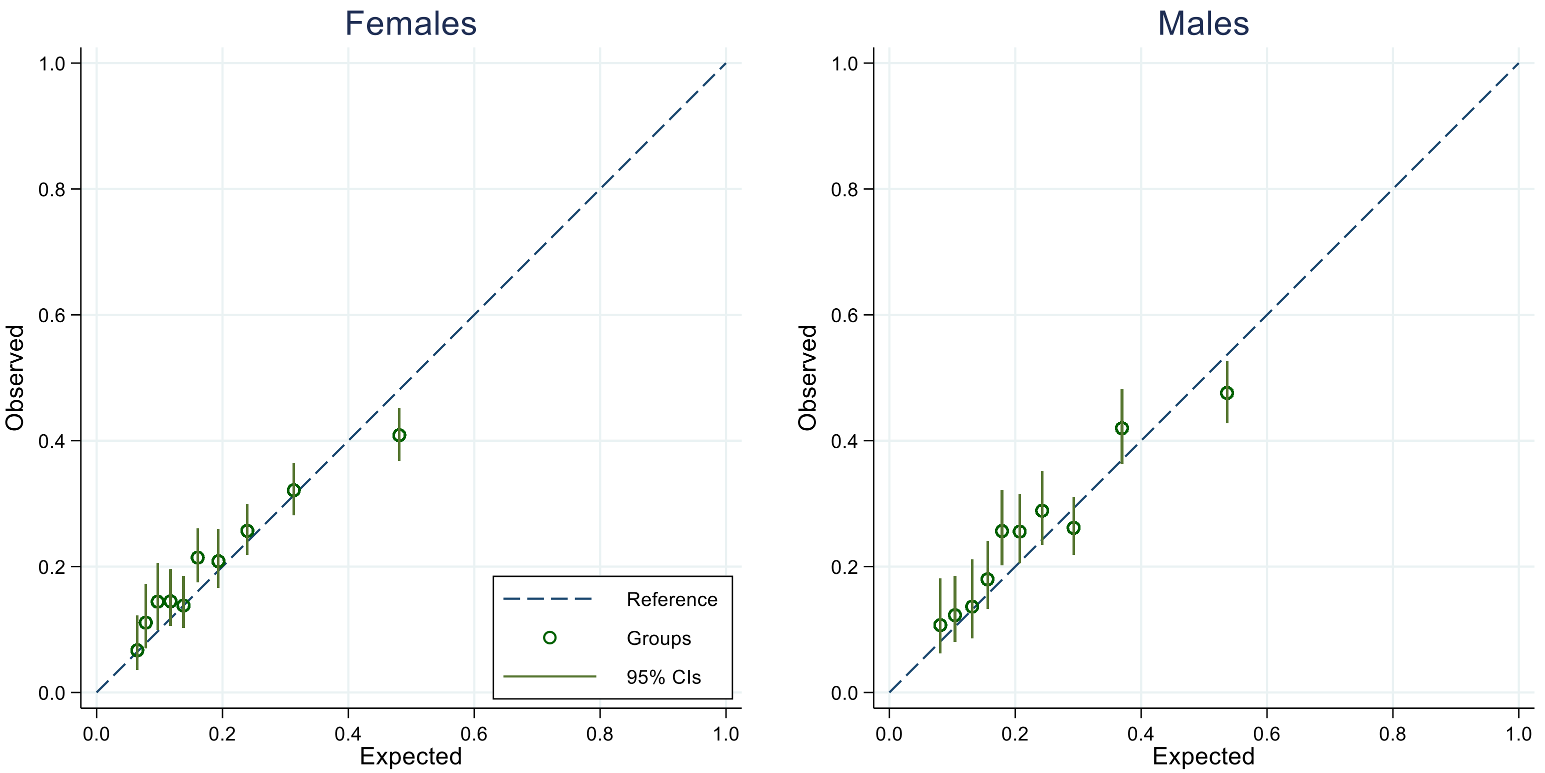

Supplementary Figure S4. Kaplan-Meier Type 2 diabetes-free probability and 95% confidence intervals for the male and female models in one of the imputed validation datasets.

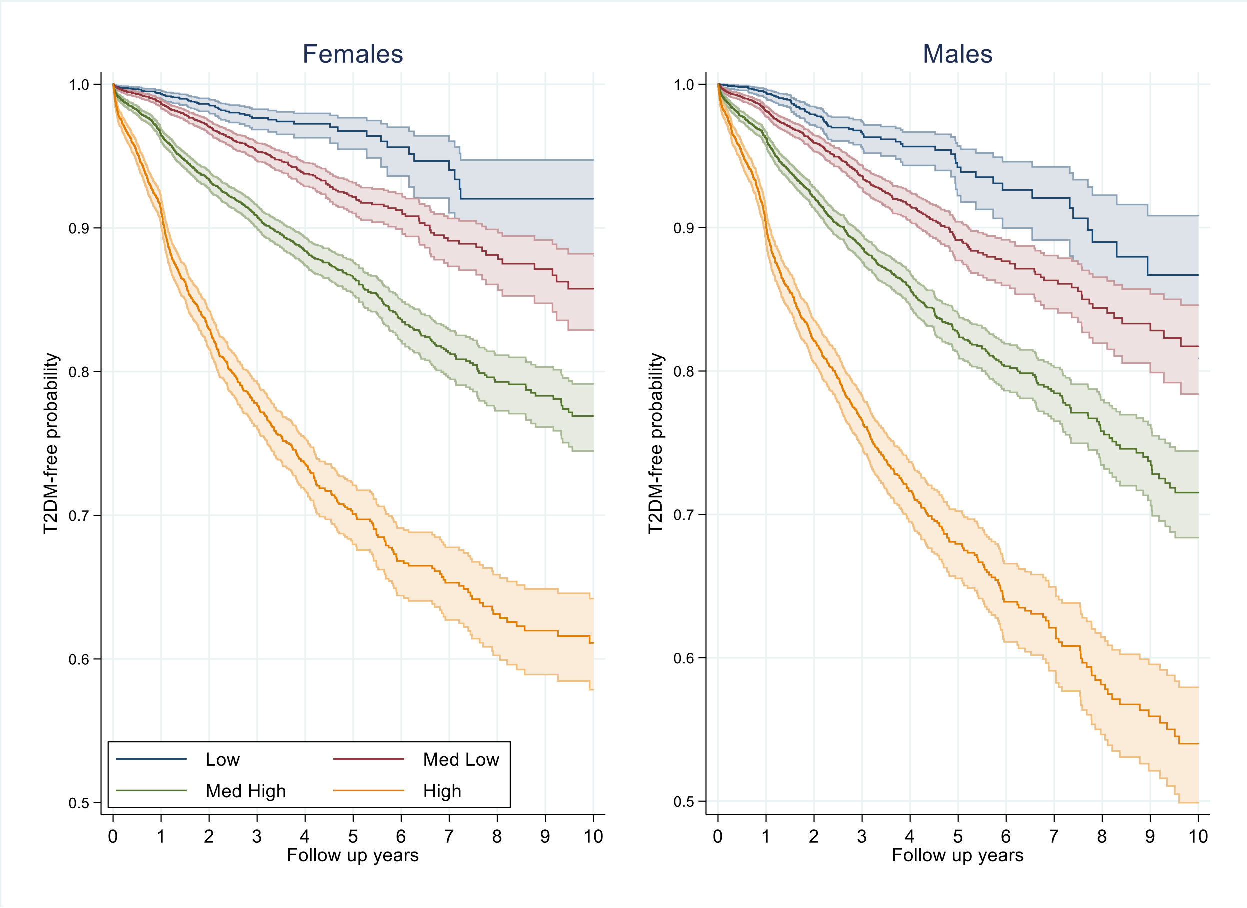

nly

The RECORD statement – checklist of items, extended from the STROBE statement, that should be reported in observational studies using routinely collected health data.

	Item No.	STROBE items	Location in manuscript where items are reported	RECORD items	Location in manuscript where items are reported
**Title and abstract**					
	1	(a) Indicate the study's design with a commonly used term in the title or the abstract (b) Provide in the abstract an informative and balanced summary of what was done and what was found		RECORD 1.1: The type of data used should be specified in the title or abstract. When possible, the name of the databases used should be included.<br><br>RECORD 1.2: If applicable, the geographic region and timeframe within which the study took place should be reported in the title or abstract.<br><br>RECORD 1.3: If linkage between databases was conducted for the study, this should be clearly stated in the title or abstract.	Pg 1-2<br><br>Pg 2<br><br>NA
**Introduction**					
Background rationale	2	Explain the scientific background and rationale for the investigation being reported			Pg 4-5
Objectives	3	State specific objectives, including any prespecified hypotheses			Pg 5
**Methods**					
Study Design	4	Present key elements of study design early in the paper			Pg 6
Setting	5	Describe the setting, locations, and relevant dates, including periods of recruitment, exposure, follow-up, and data collection			Pg 6-7

Participants	6	*(a) Cohort study* - Give the eligibility criteria, and the sources and methods of selection of participants. Describe methods of follow-up<br>*Case-control study* - Give the eligibility criteria, and the sources and methods of case ascertainment and control selection. Give the rationale for the choice of cases and controls<br>*Cross-sectional study* - Give the eligibility criteria, and the sources and methods of selection of participants<br><br>*(b) Cohort study* - For matched studies, give matching criteria and number of exposed and unexposed<br>*Case-control study* - For matched studies, give matching criteria and the number of controls per case		RECORD 6.1: The methods of study population selection (such as codes or algorithms used to identify subjects) should be listed in detail. If this is not possible, an explanation should be provided.<br><br>RECORD 6.2: Any validation studies of the codes or algorithms used to select the population should be referenced. If validation was conducted for this study and not published elsewhere, detailed methods and results should be provided.<br><br>RECORD 6.3: If the study involved linkage of databases, consider use of a flow diagram or other graphical display to demonstrate the data linkage process, including the number of individuals with linked data at each stage.	Pg 6-8, link to code lists provided on Pg 22<br><br>NA<br><br>Pg 6
Variables	7	Clearly define all outcomes, exposures, predictors, potential confounders, and effect modifiers. Give diagnostic criteria, if applicable.		RECORD 7.1: A complete list of codes and algorithms used to classify exposures, outcomes, confounders, and effect modifiers should be provided. If these cannot be reported, an explanation should be provided.	link to code lists provided on Pg 22
Data sources/ measurement	8	For each variable of interest, give sources of data and details of methods of assessment (measurement).<br>Describe comparability of assessment methods if there is more than one group			Pg 6-8

For peer review only

Bias	9	Describe any efforts to address potential sources of bias			Pg 18-19
Study size	10	Explain how the study size was arrived at			Pg 7
Quantitative variables	11	Explain how quantitative variables were handled in the analyses. If applicable, describe which groupings were chosen, and why			NA
Statistical methods	12	(a) Describe all statistical methods, including those used to control for confounding<br>(b) Describe any methods used to examine subgroups and interactions<br>(c) Explain how missing data were addressed<br>(d) *Cohort study* - If applicable, explain how loss to follow-up was addressed<br>*Case-control study* - If applicable, explain how matching of cases and controls was addressed<br>*Cross-sectional study* - If applicable, describe analytical methods taking account of sampling strategy<br>(e) Describe any sensitivity analyses			Pg 6-11
Data access and cleaning methods		..		RECORD 12.1: Authors should describe the extent to which the investigators had access to the database population used to create the study population.	Pg 6, Figure S1

For peer review only

				RECORD 12.2: Authors should provide information on the data cleaning methods used in the study.	Pg 7
Linkage		..		RECORD 12.3: State whether the study included person-level, institutional-level, or other data linkage across two or more databases. The methods of linkage and methods of linkage quality evaluation should be provided.	Pg 6<br>Linkage was not performed by the research team, rather linked data are obtained from CPRD directly
**Results**					
Participants	13	(a) Report the numbers of individuals at each stage of the study (*e.g.*, numbers potentially eligible, examined for eligibility, confirmed eligible, included in the study, completing follow-up, and analysed)<br>(b) Give reasons for non-participation at each stage.<br>(c) Consider use of a flow diagram		RECORD 13.1: Describe in detail the selection of the persons included in the study (*i.e.,* study population selection) including filtering based on data quality, data availability and linkage. The selection of included persons can be described in the text and/or by means of the study flow diagram.	Pg 5
Descriptive data	14	(a) Give characteristics of study participants (*e.g.*, demographic, clinical, social) and information on exposures and potential confounders<br>(b) Indicate the number of participants with missing data for each variable of interest<br>(c) *Cohort study* - summarise follow-up time (*e.g.*, average and total amount)			Table 2
Outcome data	15	*Cohort study* - Report numbers of outcome events or summary measures over time<br>*Case-control study* - Report numbers in each exposure			Figure S1

		category, or summary measures of exposure<br>*Cross-sectional study* - Report numbers of outcome events or summary measures			
Main results	16	(a) Give unadjusted estimates and, if applicable, confounder-adjusted estimates and their precision (e.g., 95% confidence interval). Make clear which confounders were adjusted for and why they were included<br>(b) Report category boundaries when continuous variables were categorized<br>(c) If relevant, consider translating estimates of relative risk into absolute risk for a meaningful time period			Table 3<br>Supp. Table S3 caption<br>NA
Other analyses	17	Report other analyses done—e.g., analyses of subgroups and interactions, and sensitivity analyses			NA
**Discussion**					
Key results	18	Summarise key results with reference to study objectives			Pg 12-16
Limitations	19	Discuss limitations of the study, taking into account sources of potential bias or imprecision. Discuss both direction and magnitude of any potential bias		RECORD 19.1: Discuss the implications of using data that were not created or collected to answer the specific research question(s). Include discussion of misclassification bias, unmeasured confounding, missing data, and changing eligibility over time, as they pertain to the study being reported.	Pg 13
Interpretation	20	Give a cautious overall interpretation of results considering objectives,			Pg 17-20

For peer review only

		limitations, multiplicity of analyses, results from similar studies, and other relevant evidence			
Generalisability	21	Discuss the generalisability (external validity) of the study results			Pg 20
**Other Information**					
Funding	22	Give the source of funding and the role of the funders for the present study and, if applicable, for the original study on which the present article is based			Pg 21
Accessibility of protocol, raw data, and programming code		..		RECORD 22.1: Authors should provide information on how to access any supplemental information such as the study protocol, raw data, or programming code.	Pg 22

*Reference: Benchimol EI, Smeeth L, Guttmann A, Harron K, Moher D, Petersen I, Sørensen HT, von Elm E, Langan SM, the RECORD Working Committee. The REporting of studies Conducted using Observational Routinely-collected health Data (RECORD) Statement. *PLoS Medicine* 2015; in press.

*Checklist is protected under Creative Commons Attribution (CC BY) license.

For peer review only

TRIPOD Checklist: Prediction Model Development and Validation

Section/Topic	Item		Checklist Item	Page
**Title and abstract**				
Title	1	D;V	Identify the study as developing and/or validating a multivariable prediction model, the target population, and the outcome to be predicted.	**1**
Abstract	2	D;V	Provide a summary of objectives, study design, setting, participants, sample size, predictors, outcome, statistical analysis, results, and conclusions.	**2-3**
**Introduction**				
Background and objectives	3a	D;V	Explain the medical context (including whether diagnostic or prognostic) and rationale for developing or validating the multivariable prediction model, including references to existing models.	**4-5**
	3b	D;V	Specify the objectives, including whether the study describes the development or validation of the model or both.	**5**
**Methods**				
Source of data	4a	D;V	Describe the study design or source of data (e.g., randomized trial, cohort, or registry data), separately for the development and validation data sets, if applicable.	**6**
	4b	D;V	Specify the key study dates, including start of accrual; end of accrual; and, if applicable, end of follow-up.	**6**
Participants	5a	D;V	Specify key elements of the study setting (e.g., primary care, secondary care, general population) including number and location of centres.	**6**
	5b	D;V	Describe eligibility criteria for participants.	**6**
	5c	D;V	Give details of treatments received, if relevant.	**NA**
Outcome	6a	D;V	Clearly define the outcome that is predicted by the prediction model, including how and when assessed.	**7-8**
	6b	D;V	Report any actions to blind assessment of the outcome to be predicted.	**NA**
Predictors	7a	D;V	Clearly define all predictors used in developing or validating the multivariable prediction model, including how and when they were measured.	**8-9**
	7b	D;V	Report any actions to blind assessment of predictors for the outcome and other predictors.	**NA**
Sample size	8	D;V	Explain how the study size was arrived at.	**7**
Missing data	9	D;V	Describe how missing data were handled (e.g., complete-case analysis, single imputation, multiple imputation) with details of any imputation method.	**9**
Statistical analysis methods	10a	D	Describe how predictors were handled in the analyses.	**8-9**
	10b	D	Specify type of model, all model-building procedures (including any predictor selection), and method for internal validation.	**9-10**
	10c	V	For validation, describe how the predictions were calculated.	**10-11**
	10d	D;V	Specify all measures used to assess model performance and, if relevant, to compare multiple models.	**11**
	10e	V	Describe any model updating (e.g., recalibration) arising from the validation, if done.	**11**
Risk groups	11	D;V	Provide details on how risk groups were created, if done.	**11**
Development vs. validation	12	V	For validation, identify any differences from the development data in setting, eligibility criteria, outcome, and predictors.	**10**
**Results**				
Participants	13a	D;V	Describe the flow of participants through the study, including the number of participants with and without the outcome and, if applicable, a summary of the follow-up time. A diagram may be helpful.	**Fig S1**
	13b	D;V	Describe the characteristics of the participants (basic demographics, clinical features, available predictors), including the number of participants with missing data for predictors and outcome.	**Tab 2**
	13c	V	For validation, show a comparison with the development data of the distribution of important variables (demographics, predictors and outcome).	**Sup Fig S2**
Model development	14a	D	Specify the number of participants and outcome events in each analysis.	**Fig S1**
	14b	D	If done, report the unadjusted association between each candidate predictor and outcome.	**NA**
Model specification	15a	D	Present the full prediction model to allow predictions for individuals (i.e., all regression coefficients, and model intercept or baseline survival at a given time point).	**Tab 3**
	15b	D	Explain how to the use the prediction model.	**13**
Model performance	16	D;V	Report performance measures (with CIs) for the prediction model.	**Tab 4**
Model-updating	17	V	If done, report the results from any model updating (i.e., model specification, model performance).	**19-20**
**Discussion**				
Limitations	18	D;V	Discuss any limitations of the study (such as nonrepresentative sample, few events per predictor, missing data).	**13**
Interpretation	19a	V	For validation, discuss the results with reference to performance in the development data, and any other validation data.	**Tab 4**
	19b	D;V	Give an overall interpretation of the results, considering objectives, limitations, results from similar studies, and other relevant evidence.	**18-19**
Implications	20	D;V	Discuss the potential clinical use of the model and implications for future research.	**20**
**Other information**				
Supplementary information	21	D;V	Provide information about the availability of supplementary resources, such as study protocol, Web calculator, and data sets.	**22**
Funding	22	D;V	Give the source of funding and the role of the funders for the present study.	**21**

*Items relevant only to the development of a prediction model are denoted by D, items relating solely to a validation of a prediction model are denoted by V, and items relating to both are denoted D;V. We recommend using the TRIPOD Checklist in conjunction with the TRIPOD Explanation and Elaboration document.

For peer review only - http://bmjopen.bmj.com/site/about/guidelines.xhtml
